# Homoharringtonine: updated insights into its efficacy in hematological malignancies, diverse cancers and other biomedical applications

**DOI:** 10.1186/s40001-024-01856-x

**Published:** 2024-05-04

**Authors:** Somanjana Khatua, Sudeshna Nandi, Anish Nag, Surjit Sen, Nilanjan Chakraborty, Arghya Naskar, Eda Sönmez Gürer, Daniela Calina, Krishnendu Acharya, Javad Sharifi-Rad

**Affiliations:** 1 Department of Botany, Faculty of Science, University of Allahabad, Prayagraj, Uttar Pradesh 211002 India; 2 Department of Botany, Molecular and Applied Mycology and Plant Pathology Laboratory, University of Calcutta, 35, Ballygung Circular Road, Kolkata, India; 3 Department of Life Sciences, CHRIST (Deemed to Be University), Bangalore Central Campus, Bangalore, Karnataka India; 4 Department of Botany, Fakir Chand College, Diamond Harbour, South 24-Parganas, Kolkata, India; 5 Department of Botany, Scottish Church College, Kolkata, India; 6 Department of Pharmacognosy, Faculty of Pharmacy, Sivas Cumhuriyet University, Sivas, Turkey; 7 Department of Clinical Pharmacy, University of Medicine and Pharmacy of Craiova, 200349 Craiova, Romania; 8 Facultad de Medicina, Universidad del Azuay, Cuenca, Ecuador

**Keywords:** Homoharringtonine, Leukemia treatment, Molecular mechanisms, Hematological malignancies, Pharmacological efficacy, Cancer therapeutics

## Abstract

HHT has emerged as a notable compound in the realm of cancer treatment, particularly for hematological malignancies. Its multifaceted pharmacological properties extend beyond traditional applications, warranting an extensive review of its mechanisms and efficacy. This review aims to synthesize comprehensive insights into the efficacy of HHT in treating hematological malignancies, diverse cancers, and other biomedical applications. It focuses on elucidating the molecular mechanisms, therapeutic potential, and broader applications of HHT. A comprehensive search for peer-reviewed papers was conducted across various academic databases, including ScienceDirect, Web of Science, Scopus, American Chemical Society, Google Scholar, PubMed/MedLine, and Wiley. The review highlights HHT's diverse mechanisms of action, ranging from its role in leukemia treatment to its emerging applications in managing other cancers and various biomedical conditions. It underscores HHT's influence on cellular processes, its efficacy in clinical settings, and its potential to alter pathological pathways. HHT demonstrates significant promise in treating various hematological malignancies and cancers, offering a multifaceted approach to disease management. Its ability to impact various physiological pathways opens new avenues for therapeutic applications. This review provides a consolidated foundation for future research and clinical applications of HHT in diverse medical fields.

## Introduction

Blood cancers, a diverse collection of malignancies impacting the blood, bone marrow, and lymphatic system, are marked by the uncontrolled growth of lymphocytes, myeloid cells, or various blood cell precursors. This group includes various diseases, such as leukemias, lymphomas, and multiple myeloma, each with its distinct genetic, molecular, and clinical characteristics [1]. Globally, hematological cancers represent a significant health burden with variable incidence across different populations [2]. Leukemia, a blood-related malignancy characterized by transformed hematopoietic progenitors and diffused infiltration of bone marrow, poses a significant health burden globally, affecting both developed and developing countries [3].

Leukemia is mainly categorized into four key types, distinguished by the primary cell type involved (myeloid or lymphoid) and the speed of disease advancement (acute or chronic). These types are chronic myeloid leukemia (CML), acute myeloid leukemia (AML), chronic lymphocytic leukemia (CLL), and acute lymphocytic leukemia (ALL). Notably, CML and AML account for about half of all new leukemia diagnoses worldwide [4, 5].

CML, a slow-growing leukemia, accounts for about 15% of newly diagnosed cases in adults [6]. It is primarily characterized by forming the Philadelphia (Ph) chromosome, which encodes the p210BCR–ABL oncoprotein. This protein exhibits constitutive tyrosine kinase activity, inhibiting apoptosis and promoting independent growth [7]. In the 1980s, interferon (IFN)-α was the frontline treatment for patients in the chronic phase (CP) of CML. The landscape of medical therapy for this disease was revolutionized with the development of the tyrosine kinase inhibitor (TKI), imatinib mesylate. This medication has been shown to yield a 98% complete hematologic response (CHR) and an 83% major cytogenetic response (MCyR) in newly diagnosed CP CML patients. However, resistance to this drug, primarily due to BCR–ABL mutations, remains a significant challenge, highlighting the need for additional non-cross-resistant therapies [8].

AML presents an even more daunting challenge, accounting for the highest incidence rate among leukemias and contributing to the highest rate (62%) of leukemic deaths [9]. The standard treatment, dating back to the 1970s, involves cytotoxic chemotherapy aimed at impairing nucleic acid synthesis. This regimen, known as the “3 + 7 regimen,” combines continuous 7-day administration of cytarabine (ara-C) with short infusions of an anthracycline during the first 3 days. Although this treatment can be effective, it is often poorly tolerated, especially by elderly, relapsed/refractory, and secondary AML patients, underscoring the need for a milder, yet highly effective and sensitive chemotherapeutic regimen with reduced toxicity [10].

In this context, HHT, a plant-derived alkaloid [Cephalotaxine, 40-methyl (20R)-hydroxy-20-(400-hydroxy-400-methylpentyl)-butanedioate (ester), [3(R)]-(9CI)] has been recognized for its antitumor properties. Used extensively in China for over 40 years, HHT has shown efficacy in treating hematological malignancies, including CML, AML, and myelodysplastic syndrome (MDS) [11]. Substantial research in the United States has also confirmed its effectiveness against CML [12]. HHT's anti-leukemic mechanism involves inhibiting the translation process by affecting the A site in the ribosome, leading to rapid loss of several short-lived proteins crucial for cell survival, such as myeloid cell leukemia (MCL)-1, cyclin D1, the pro-oncoprotein c-Myc, and the X-linked inhibitor of apoptosis. This process triggers cell death. HHT also targets the phosphorylated serine 209 residues of the eukaryotic translation initiation factor eIF4E, resulting in the degradation of the phosphorylated protein and hindering the growth of leukemia cells both in-vitro and in-vivo [13]. In addition, HHT has been shown to reduce the expression level of the p210BCR‑ABL protein in BCR‑ABL + cells, thus emerging as an effective treatment for CML patients who are unresponsive to IFN-α therapy [7]. Notably, HHT has led to remission in 92% of treated patients, with a cytogenetic response observed in 68% [15]. The development of a semisynthetic purified HHT compound, omacetaxine mepesuccinate (OM), has further enhanced its clinical utility. OM's effectiveness in recent studies has led to its approval by the American Food and Drug Administration (FDA) on October 26, 2012, for the treatment of CP or accelerated phase (AP) CML after failure of two or more TKIs, thus garnering widespread attention for HHT [14].

Recent research has also indicated a synergistic relationship between OM and HHT when combined with ara-C, imatinib, or IFN-α in clinical therapy [7, 12]. Current investigations are thus directed towards augmenting therapeutic prospects by combining the pure compound with other treatments in low dosages, particularly in patients with worse prognoses [15].

Furthermore, HHT has been identified as an active anticancer agent in a spectrum of disorders, including AML, MDS, acute promyelocytic leukemia, polycythemia vera, and as an intrathecal cure for central nervous system leukemia [14]. Its inhibitory effects on solid tumor cells, such as lung, breast, liver, colorectal, and skin cancer, have also been documented [16–18]. Beyond its anticancer effects, HHT has been found to possess a range of beneficial properties, including anti-inflammatory, antiviral, antimicrobial, anti-diabetic, anti-allergic, and neuroprotective properties [13, 19, 20]. Recent publications have highlighted the anti-leukemic effects of HHT [14, 21], but a comprehensive aggregation of all data on its biological effects remains to be synthesized. Despite advancements in diagnosis and treatment, hematological malignancies remain a therapeutic challenge due to their complexity and the frequent development of resistance to current therapies; in this landscape, HHT has emerged as a promising agent. This paper reviews updated insights into the efficacy of HHT in treating various hematological malignancies, solid tumors, and its potential applications in other biomedical areas. By delineating the molecular mechanisms of HHT and its clinical outcomes, it aims to underscore its role in the evolving paradigm of targeted cancer therapy.

## Review methodology

For this comprehensive review, an extensive search for peer-reviewed papers was carried out across a wide range of academic databases: ScienceDirect, Web of Science, Scopus, American Chemical Society, Google Scholar, PubMed/MedLine and Wiley. To streamline the search process, a strategic combination of keywords and phrases was employed.; including specific terms, such as “leukemia,” “chronic myeloid leukemia,” “treatment strategy in CML,” “acute myeloid leukemia,” “treatment strategy in AML,” “HHT,” “HHT mode of action,” “HHT combined regimen,” “HHT structure”, “HHT antiviral effect”, “HHT bioactivity”, and “HHT anti-inflammatory effect”. In addition, Boolean operators such as “AND,” “OR,” and “NOT” were used to refine and expand the literature search, ensuring a comprehensive collection of relevant research articles and review articles. The selection criteria focused on the innovativeness of the studies, the clarity and systematic nature of their explanations, and their impact on the scientific community.

The inclusion criteria for selecting these manuscripts were primarily based on their relevance to the specific topics of interest and their contribution to advancing our understanding of HHT; published articles that offered innovative insights, systematic reviews, or were of high impact in the field were given priority. Exclusion criteria were applied to filter out studies that were not directly relevant to the keywords, lacked substantial novelty or systematic analysis, or did not significantly contribute to the field’s understanding mechanisms of HHT. The chemical formulas have been validated according to PubChem [22], and taxonomy of the plant has been validated according to World Flora Online [23]. The most representative data are summarized in tables and figures.

## A brief overview on HHT: from natural origins to synthetic derivatives—structural, physicochemical and biosynthetic aspects

### Natural sources and traditional uses of HHT

Plants have long been the most significant source of potential bioactive natural compounds for obvious novel reasons. In nature, harringtonine and HHT are present in the coniferous tree *Cephalotaxus* commonly known as Plum Yew or Cowtail Pine which is the sole member of the family Cephalotaxaceae [24]. The genus having at least 9 species are found in Asia, concentrated mostly in China, but are also reported from Thailand, India, Japan and the Korean peninsula [21, 25]. The genus consists of evergreen, mostly dioecious, occasionally monoecious shrubs or small trees thriving in rich, humic soils in damp subtropical or mild temperate forests. These slow-growing conifers feature dark olive to black–green leaves. Their branches are arranged in whorls or oppositely. The buds, ovate in shape, are encased in numerous, overlapping, persistent scales. The needle-like leaves, spirally arranged on the terminal shoots but angled at the base to appear in two flat rows on side shoots, are linear, 4–12 cm long and 3–4 mm wide, with a soft texture and blunt end. Male cones are spherical and axillary, forming in flat-topped clusters of several small anther clusters, about 1 cm in diameter, and positioned in the axils along the branchlets. Female cones are grouped in clusters of 6–12 ovules, in pairs at the axils of terminal bud scales, hanging down, drupe-like, elliptical, 2–3 cm long, with a leathery, fleshy exterior. In general, one seed ripens per cone, with 3–5 female cones growing on stalks at or near the tip of the current or previous year's shoots. Female cones are wind-pollinated. The seeds lack wings and undergo a relatively extended development period [26–28]. The genus having eight species from which two different groups of alkaloids viz. cephalotaxine and homoerythrina are obtained and comprise 28 different compounds. Cephalotaxine series contains 18 of those compounds, whereas homoerythrina series comprises 10 of those compounds belong to the class of alkaloid. HHT is a Cephalotaxine series of alkaloid obtained from leaves, bark and seeds of different species of *Cephalotaxus,* such as *C. harrintonia, C. hainanensis, C. qinensis, C. fortunei, and C. griffithii* [25, 29].

The seeds of this coniferous genus are toxic to humans but were used in ancient Chinese medicine. In the 1970s, collaborative research by Chinese and American scientists led to the extraction of active compounds from the bark of *Cephalotaxus*. Chinese researchers, through various screening tests and initial clinical trials, found that the total alkaloids from C. fortunei showed significant anti-cancer properties. Both water-based and later alcohol-based extracts of the seeds from *Cephalotaxus harringtonia* var drupacea demonstrated effectiveness against leukemia cells when tested in vitro. Powell and his team conducted a detailed separation of the seed extract from this particular variety and other types of *Cephalotaxus*. Through this process, they were the first to describe the structure of cephalotaxine and related alkaloids, which were found to lack biological activity [30]. Subsequently, in 1972, Powell and his team published findings on the anti-cancer properties of extracts from various *Cephalotaxus* species. They isolated a series of cephalotaxine esters, including harringtonine, isoharringtonine, HHT, and doxyharringtonine. Among these, HHT was identified as the most effective in extending the survival of mice with P388 leukemia [29]. Recently, to address the substantial market demand for HHT, extensive research has been conducted to identify endophytic fungi in *Cephalotaxus* that produce HHT. A variety of endophytic fungi associated with *Cephalotaxus* have been discovered, from which several bioactive compounds such as anthraquinone, sesquiterpenoids, and aromatic metabolites have been isolated. These compounds exhibit cytotoxic and antimicrobial properties. However, there has been no report of any endophytic fungus capable of synthesizing HHT [31–35]. Hu et al. for the first time reported HHT producing endophytic fungi from the bark of *C. hainanensis* and the fungus was identified as *Alternaria tenuissima* [36].

### Structural features of HHT

**Fig. 1:**
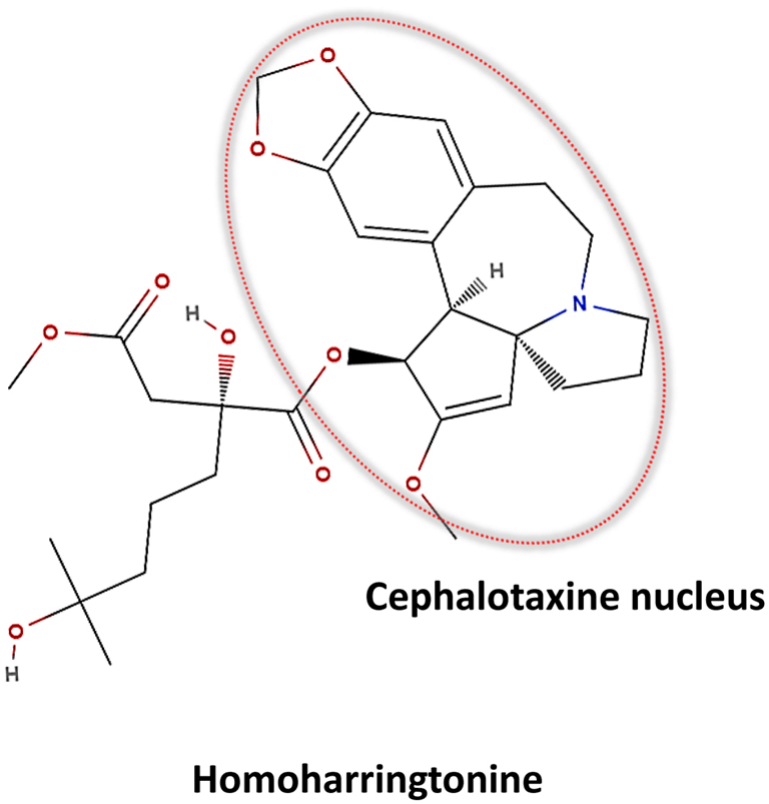
Molecular structure of HHT highlighting the cephalotaxine core. The figure presents the chemical structure of HHT with the cephalotaxine nucleus central to its molecular architecture outlined in red; key functional groups are depicted, including the ester linkages (highlighted in red) and the nitrogen-containing heterocycle (marked in blue), which are important for HHT's pharmacological activity

HHT (NSC no 141633, 4-methy-2-hydroxy-4-methylpentyl butanedioate), a polycyclic alkaloid, is the alkyl-substituted succinic acid ester derivative of another alkaloid (–)-Cephalotaxine [12] (Fig. 1).

HHT has a methylene group in its side chain and differs from its bio-inactive parent Cephalotaxine. The functional groups in the compound are arene, benzene ring, alkene, amine, amine, tertiary, acetal, alkanol, carbonyl, enol ether, ester (carboxylate ester), ether [37].

### Physio-chemical properties of HHT

HHT has a molecular formula C_29_H_39_NO_9_. Structurally, it can assume two forms such as amorphous and crystalline. The amorphous HHT was reported to be more soluble in water when compared with its crystalline counterpart. Furthermore, at 95% relative humidity, crystalline HHT was more prone to structural alternation [38]. Furthermore, force degradation of HHT to the acidic and alkaline stress showed that degradation of HHT followed different paths, such as linkage hydrolysis of 1,3-dioxole ring, N-oxide derivatives, hydrolysis of methyl ester etc. [39]. LC–MS characterization revealed the exact mass of HHT as 545.26251 g/mol (https://massbank.eu/MassBank). The top four peaks in the positive mode LC–ESI–ITFT are 546.2696, 999, 298.1435 and 30. Furthermore, Hu et al. [36] reported LC/MS molecular ions of HHT as m/z 298.2 [M–C_11_H_19_O_6_]^+^, m/z 266.4 [M–C_11_H_19_O_6_–CH_3_O]^+^, m/z 251.3 [M–C_11_H_19_O_6_–CH_2_O_2_]^+^, m/z 240.4 [M–C_11_H_19_O_6_–C_3_H_5_O]^+^, and m/z 226.3 [M–C_11_H_19_O_6_–C_4_H_5_O]^+^. Previously, similar LC–MS spectra were reported by Choi et al. [40].

### Biosynthesis of HHT

**Fig. 2:**
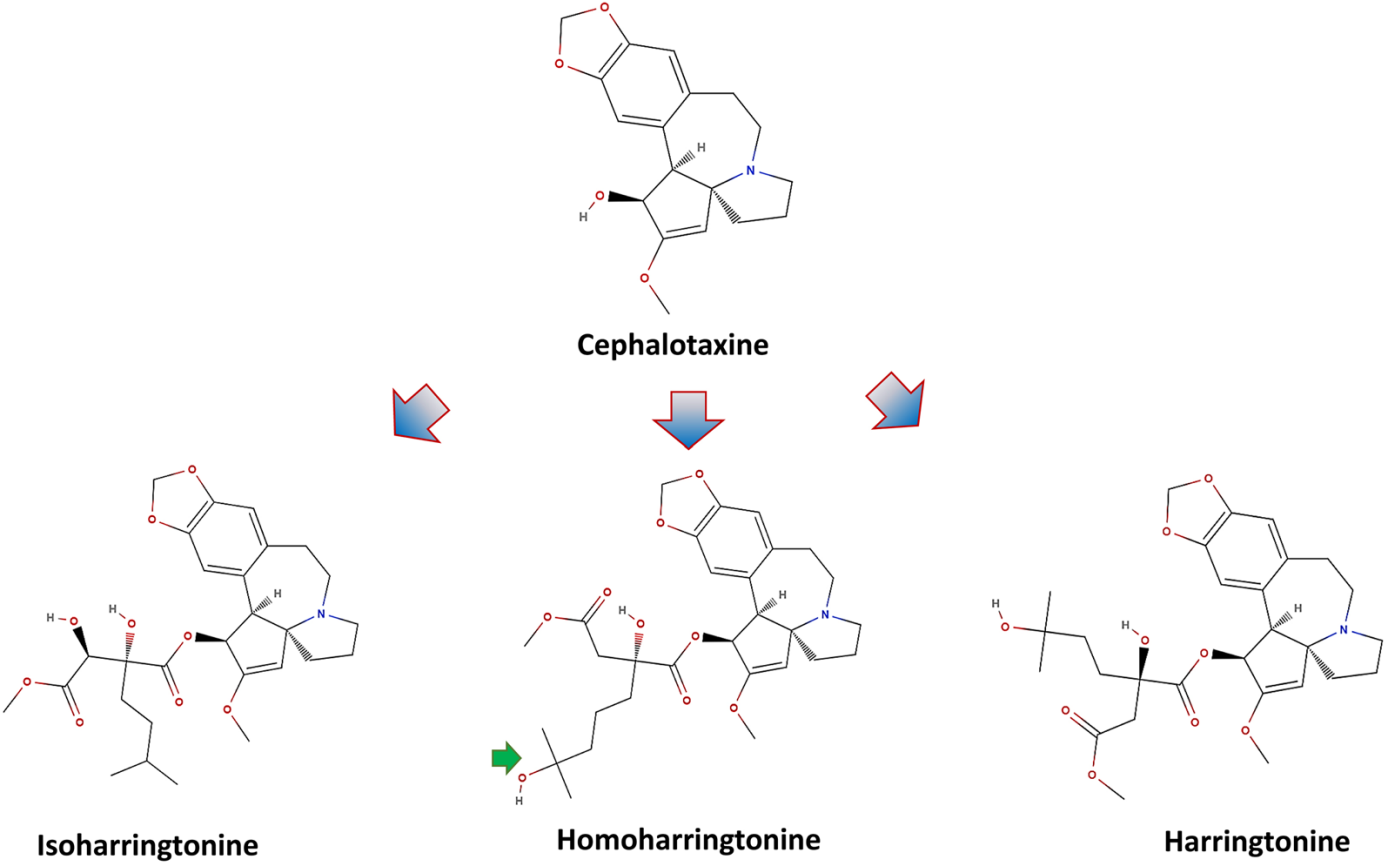
Comparative molecular structures of cephalotaxine and its derivatives. The figure displays the chemical structures of cephalotaxine and its notable derivatives: isoharringtonine, homoharringtonine, and harringtonine. Central to each molecule is the cephalotaxine core, denoted by the blue arrows, which serves as the foundational scaffold for these compounds. Homoharringtonine is specifically marked with a green check to highlight its clinical significance

**Fig. 3:**
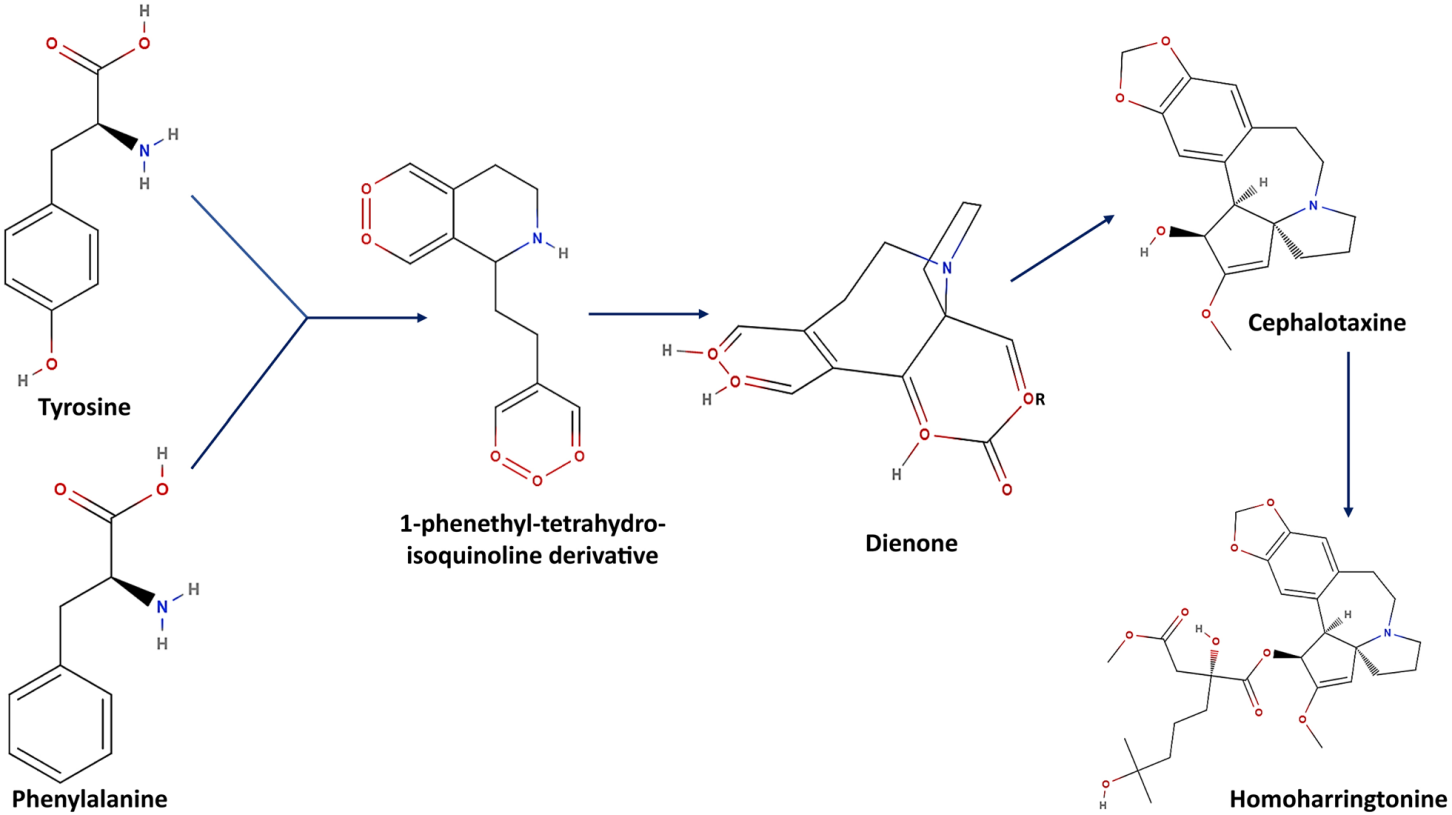
Biosynthetic pathway of HHT. The schematic diagram outlines the biosynthesis of HHT from tyrosine and phenylalanine. Initial steps form a 1-phenethyl-tetrahydroisoquinoline derivative, which transforms into a dienone and then the cephalotaxine skeleton; the final step results in HHT, emphasizing the compound's complex natural production [42]

The biosynthesis pathway of HHT is not yet understood completely. However, a few attempts have been made to elucidate the biosynthesis of HHT using isotope labelling. Nett et al. [41] utilized the D2O labelling technique to comprehend the HHT biosynthesis in the needle of *C. harringtonia*. Roughly, it was found that the cephalotaxine core of HHT was derived from two amino acids, namely tyrosine and phenylalanine. These two amino acids contribute to forming the skeleton via ring contraction of l-phenethyl-tetrahydro-isoquinoline derivative and a dienone. Specifically, it was hypothesized that C-6, C-7, and C-8 side chain of Cephalotaxine was synthesized from C-3, C-2, and C-1 side chains of phenylalanine, respectively. Similarly, C-10 and C-11 of the skeleton were sourced from C-2 and C-3 of the tyrosine side chain, respectively. Finally, the esterification of the side chain is naturally done by a chain elongation mechanism similar to the Leucine biosynthesis pathway [42] (Figs. 2 and 3).

### Synthetic derivatives of HHT

As mentioned earlier, HHT is in general derived synthetically from the plant alkaloid Cephalotaxine. However, several methodologies for the chemical synthesis of Cephalotaxine as an immediate precursor of HHT are recorded in the literature. In the seventies, early synthesis of Cephalotaxine was reported. Semmelhack et al. [43] synthesized Cephalotaxine using p nitrobenzenesulfonate ester as a precursor compound. Similarly, Weinreb and Auerbach [44] produced Cephalotaxine in eight steps from 1-prolinol and 3,4 methylenedioxyphenylacetyl chloride. Later, Gouthami et al. [45] synthesized it with a 10% yield using an aryne insertion reaction as a critical step. Ju and Beaudry [46] developed a novel technique for the chemical synthesis of HHT. They showed a complete nine-step synthesis of Cephalotaxine and HHT, involving oxidative furan opening, transannular Mannnich and Noyori reduction reactions. Recently, Dang et al. [47] reacted an oxo-containing α-tetrasubstituted lactone with Cephalotaxine and synthesized HHT, with a high yield. Synthetic analogs of HHT, specifically Homoharringtonine 2 and Dehydrodesoxy Homoharringtonine 4, have been synthesized through a process of partial esterification of Cephalotaxine [48].

Table 1 summarizes the diverse synthetic methodologies for HHT and cephalotaxine, detailing the precursor-intermediates used, the end products synthesized, and the yields where available; consolidates various synthetic approaches towards HHT and its precursors, highlighting the evolution of synthesis techniques.

**Table 1:** Synthesis methodologies for Cephalotaxine and HHT derivatives

Methodology	Precursor-intermediate	Synthesized compound	Yield	Refs.
p-Nitrobenzenesulfonate ester reaction	p-Nitrobenzenesulfonate ester	(−) Cephalotaxine	N/A	[43]
Eight-step synthesis from 1-Prolinol	1-Prolinol methylenedioxyphenylacetyl chloride	(−) Cephalotaxine	N/A	[44]
Aryne insertion reaction	Aryne	(−) Cephalotaxine	10%	[45]
Nine-step oxidative furan opening	Various intermediates including furan derivatives	HHT	N/A	[46]
Lactone reaction with cephalotaxine	α-Tetrasubstituted lactone	HHT	High	[47]
Partial esterification	Cephalotaxine	Homoharringtonine 2 Dehydrodesoxy Homoharringtonine 4	N/A	[48]
Alkylidene Carbene 1,5-CH insertion reaction	Alkylidene carbene	(−) Cephalotaxine	N/A	[49]
Palladium-Catalyzed Enantioselective Tsuji Allylation	Allyl enol carbonate	(−) Cephalotaxine	N/A	[50]
Ester Enolate Claisen rearrangement	α-Amino allylic esters	(−) Cephalotaxine	N/A	[50]
Gold(I)-catalyzed cascade reaction	Norhydrastinine	DemethylCephalotaxinone, Cephalotaxine	N/A	[33]
Facile stevens rearrangement	Weinreb amide	( ±) cephalotaxine	N/A	
Hydrogenation	β-Substituted itaconic acid monoesters	HHT	N/A	[51]

N/A: Not Available

## HHT’s anticancer effects in hematological malignancies

### Chronological development and clinical advancement of HHT

**Fig. 4:**
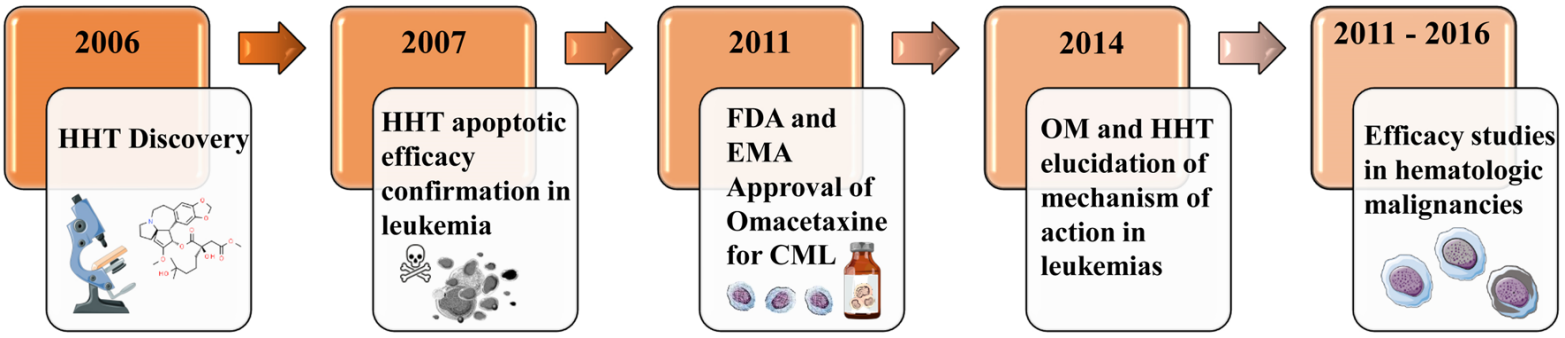
Developmental timeline of HHT from its discovery in 2006 to pivotal studies conducted through 2016. Key events include the discovery of HHT, validation of its apoptotic effects in leukemia, regulatory approvals, understanding of its action mechanism, and the span of efficacy studies in various hematologic cancers. HHT: Homoharringtonine; FDA: Food and Drug Administration; EMA: European Medicines Agency; CML: Chronic Myeloid Leukemia; OM: Omacetaxine (a semi-synthetic form of HHT used for treatment)

The journey of HHT in the realm of cancer therapeutics commenced shortly after its discovery (Table 2, Fig. 4), with preclinical studies highlighting its broad cytotoxic properties, especially against rapidly proliferating cells of both lymphoid and myeloid origins [52]. These initial findings propelled the commencement of preclinical trials in China, targeting patients with leukemic disorders. This endeavor set the stage for subsequent clinical trials in the United States, further elucidating HHT's mechanism of action. Research demonstrated that HHT exerts its anticancer effects primarily through the inhibition of the protein translation process. Omacetaxine mepesuccinate (OM), a semi-synthetic derivative of HHT, was found to bind to the A-site cleft of the peptidyltransferase center in the large subunit of the eukaryotic ribosome. This binding disrupts the correct positioning of amino acid side chains of incoming aminoacyl-tRNAs, effectively inhibiting the initial elongation step of protein synthesis, a process that is vital for cancer cell survival and proliferation [53]. The capacity of HHT to induce apoptosis was confirmed in a variety of tumor types, including leukemia, lymphoma, neuroblastoma, carcinoma, and specifically in primary leukemic cells from AML patients [54]. However, the progress in HHT research was initially hampered by the lack of a consistent source of purified HHT. To overcome this hurdle, ChemGenex in the USA developed a semi-synthetic form of HHT, initially named OP1384. This compound was later acquired by Cephalon and rebranded as CEP-41443. In 2011, following the merger of Cephalon with Teva Pharmaceuticals, OM, commercially known as Synribo with a purity of 99.7%, was introduced to the market. The European Medicines Agency (EMA) and the Food and Drug Administration (FDA) approved the subcutaneous use of OM for treating adult CML patients in chronic or accelerated phases who were intolerant or resistant to two or more tyrosine kinase inhibitors (TKIs) [53]. Subsequent studies have focused on elucidating the efficacy of omacetaxine; these investigations revealed that OM can induce the rapid degradation (in less than 4 h) of several short-lived proteins crucial for cancer cell survival, such as Mcl-1, Cyclin D1, c-Myc, XIAP, β-Catenin, and the long isoform of cellular FLICE inhibitory protein (c-FLIPL), across various cell lines derived from CML, AML, CLL, and multiple myeloma patients [55]. In addition, HHT was found to up-regulate the expression of myosin-9, a skeletal contractile protein, thereby enhancing the sensitivity of leukemia cells to the alkaloid in both AML and CML cell lines [56].

**Table 2:** Key milestones in HHT’s clinical development: a chronological overview

Years	Milestone	Description	Significance	References
2006	Initial Discovery and Preclinical Studies	Identification of HHT’s cytotoxic properties against lymphoid and myeloid cells	Foundation for the clinical potential of HHT	[52]
2007	Confirmation of Apoptotic Efficacy	HHT shown effective in various tumors, including primary leukemic cells from AML patients	Broadened therapeutic scope of HHT	[54]
2011	Regulatory Approval of Omacetaxine (OM)	OM, a semi-synthetic HHT derivative, approved by EMA and FDA for CML treatment	Marked HHT's entry into clinical use for CML	[53]
2011–2016	Advanced Efficacy Studies	Studies highlighted OM’s mechanism in rapid protein degradation and the up-regulation of myosin-9	Reinforced HHT’s effectiveness in hematologic malignancies	[55, 56]
2014	Elucidation of Mechanism of Action	Detailed understanding of how HHT and OM inhibit protein translation in cancer cells	Clarified the molecular basis of HHT's anticancer action	[53]

AML: Acute Myeloid Leukemia; CML: Chronic Myeloid Leukemia; EMA: European Medicines Agency; FDA: Food and Drug Administration; HHT: Homoharringtonine; OM: Omacetaxine Mepesuccinate

### The impact of HHT on AML treatment

#### Disease overview

Acute myeloid leukemia (AML), also referred to as acute myeloblastic leukemia, acute granulocytic leukemia, or acute nonlymphocytic leukemia, arises from leukemia stem cells or precursor cells. It is among the most prevalent and deadly types of blood cancers. AML is marked by a proliferation of myeloid cells in the bone marrow and an interruption in their maturation process. This frequently results in hematopoietic insufficiency, which can manifest with or without an increase in white blood cells [57]. It is the most common form of acute leukemia among adults accounting for approximately 70% of acute leukemias with a survival of only a few weeks to a few months, if untreated [58]. Even considering the present-day therapies, a high proportion of AML patients cannot survive beyond 5 years. Indeed, genomically heterogeneous AML has a trend to evolve, mostly under selective treatment pressure contributing varied responses to the curing process [59]. Among the different sub-types of AML, t(8;21) translocation is one of the most frequent chromosome abnormalities in AML. The chromosomal aberration results in the fusion of *AML1* gene (also called RUNX1) on chromosome 21 with *ETO* gene (also referred to as *RUNX1T1* that encodes CBFA2T1 protein) on chromosome 8 [60]. Protein product of the translocation, AML1–ETO, blocks hematological cell differentiation and thus plays a principal pathogenic role in t(8;21) leukemia [61]. Alongside, Fms-related tyrosine kinase 3 (FLT3) mutations have also been evident in the disease encompassing almost 15–25% of all AML, with higher prevalence in younger patients (≤ 60 years). About 75% of FLT3 mutations possess internal tandem duplication mutation and thus are called ITD subtype. The remaining 25% of FLT3 mutations are TKD subtypes including point mutation in the tyrosine kinase domain [62]. Both types induce ligand-independent auto-phosphorylation and stimulation of receptors leading to aberrant overexpression of a set of oncogenes, such as *MYC*. The mode of action is mediated through constitutive activation of PI3k/Akt/mTOR and MEK/ERK pathways, as well as STAT5. As a result, autonomous cell proliferation is augmented playing a key role in the pathogenesis of AML [63]. During the early 70 s, the treatment of AML included induction chemotherapy followed by consolidation or allo-HSCT. However, the recovery time of myelosuppression triggered by chemotherapy is time-consuming and most of the patients died of diverse progression or toxicity of repeated but futile chemotherapy [64]. At the present day, the contemporary treatment paradigm employs remission-inducing chemotherapy and certain inhibitors. The conventional induction therapy for AML is an anthracycline-based “3 + 7” regimen denoting a 3-day anthracycline (generally 45–60 mg/m^2^ daunorubicin or DNR or 12 mg/m^2^ idarubicin) and 1 week 100–200 mg/m^2^ cytarabine as a constant infusion ensuring long-term remedies of 30 to 40% younger patients with AML [65]. However, anthracycline-associated cardiac toxicity delineates the use, particularly in elderly patients with cardiac disease. Disease relapse and drug resistance are still two key impediments in long-term remission and overall survival of patients. Finding new novel available strategies with high efficiency that might overcome multidrug resistance and decrease toxicities is therefore urgently required for AML therapy [10].

### Pre-clinical studies

In this context, HHT has been used in China for more than 40 years to treat AML, although concomitant research published till date is limited [66]. The finding reported by Zhou et al. [67] was one of the early investigations, where HHT predominantly inhibited protein synthesis in HL-60 cells and showed a synergistic effect with l-β-D-arabinofuranosylcytosine (an inhibitor of DNA synthesis). Later on, several experiments have shown that the plant alkaloid can accelerate apoptosis in a range of AML cells, possibly by enhancing Bax expression and has no apparent cross-resistance to medications like DNR and cytarabine [68]. A significant reduction in cell proliferation of KG1, a human AML cell line, has also been noticed when challenged with HHT [10]. It also demonstrated effectiveness in enhancing MCL-1 degradation, leading to mitochondrial disruption and the release of Cytochrome C, which in turn activates caspases in the HL60 and HL60/MRP myeloid leukemia cell lines [69].

The idea of synergistic interactions between drugs has been an essential concern in the biomedical world. In this context, HHT presented additive effects with other chemotherapeutic drugs as reported in several prior studies. For instance, HHT exerted a synergistic anti-leukaemia effect with venetoclax by decreasing mitochondrial membrane potential (MMP) (ΔΨm), reducing expression of Mcl-1 protein, inhibiting PI3k/Akt and MAPK/ERK pathways, activating p53 pathway, arresting cell cycle progression and inducing apoptosis in AML cells.

Moreover, the combination also inhibited AML progression and significantly prolonged survival time in xenograft mice [70]. HHT and oridonin (a compound derived from herb, *Isodon rubescencs*) have also been shown to exert synergistic effects in AML cell line viability. The combination triggered MMP loss, downregulation of Mcl-1, decrease in total and phosphorylated c-KIT protein levels, and increase in caspase-3 activation and induction of apoptosis. Besides, HHT elevated intracellular levels of oridonin enhancing the overall effect. The combination also prolonged t(8;21) leukemia mouse survival suggesting its probable use as an effective therapy [61]. Wang et al. [71] reported that combining HHT with aclarubicin can synergistically suppress PI3k/Akt and WNT/β‐catenin signalling in AML cells. In another study, HHT together with aclarubicin and cytarabine (HAA) was validated as a highly effective treatment for AML, especially for t (8;21). The regimen synergistically promoted apoptosis in leukemia cells inducing caspase-3-mediated cleavage of AML1–ETO oncoprotein [72]. In contrast, He et al. [68] advocated that HHT in combination with cytarabine and etoposide (HAE) regimen might function as an effective and tolerable program for first-line treatment of AML. HHT and etoposide exhibited synergistic cytotoxicity in AML cell lines and primary AML cells mediated through the increase of reactive oxygen species (ROS) synthesis and restricting thioredoxin-mediated antioxidant defence [73]. HHT alone or in combination exhibited a potent cytotoxic effect against FLT3–ITD positive cell lines and primary leukemia cells [74]. Li et al. [75] performed an extensive study showing that HHT and ibrutinib possess high sensitivity towards FLT3–ITD positive cells than wild type. The drugs in combination enhanced cell cycle arrest and apoptosis in MV4-11 and MOLM-13 leukemia cells indicating a synergistic effect. Further elucidation of the mechanism unveiled that the effect was mainly through regulating STAT5/Pim-2/C-Myc pathway which was concomitant with in vitro studies. In another study, quizartinib (also known as AC220) and HHT exhibited a synergistic effect to decrease FLT3–ITD cell viability. Mechanistically, both of them cooperatively arrested the cell cycle at the G1 phase [76]. The combination of Venetoclax (also known as ABT-199) and HHT effectively hindered the proliferation of AML cells with the FLT3–ITD mutation by inducing apoptosis in a dose- and time-dependent manner. The underlying mechanism involved the suppression of anti-apoptotic proteins such as Bcl-2 and MCL-1 and the activation of caspases, including caspase-3 and caspase-9. In addition, the cell cycle was arrested, inhibiting DNA synthesis and thereby reducing cell proliferation, which contributed to the anti-leukemic effects. Notably, the study found that this combination could significantly reduce the expression of p-FLT3 and its downstream signaling proteins, such as p-Stat5 and MCL-1, leading to apoptosis in AML cell lines. Furthermore, this treatment also suppressed the growth and progression of AML cells in an in vivo xenograft model [77]. The combined cytotoxic effect of ABT-199 and HHT was further corroborated in vivo using mouse xenograft models with primary cells derived from Diffuse Large B-Cell Lymphoma (DLBCL) [78].

**Fig. 5:**
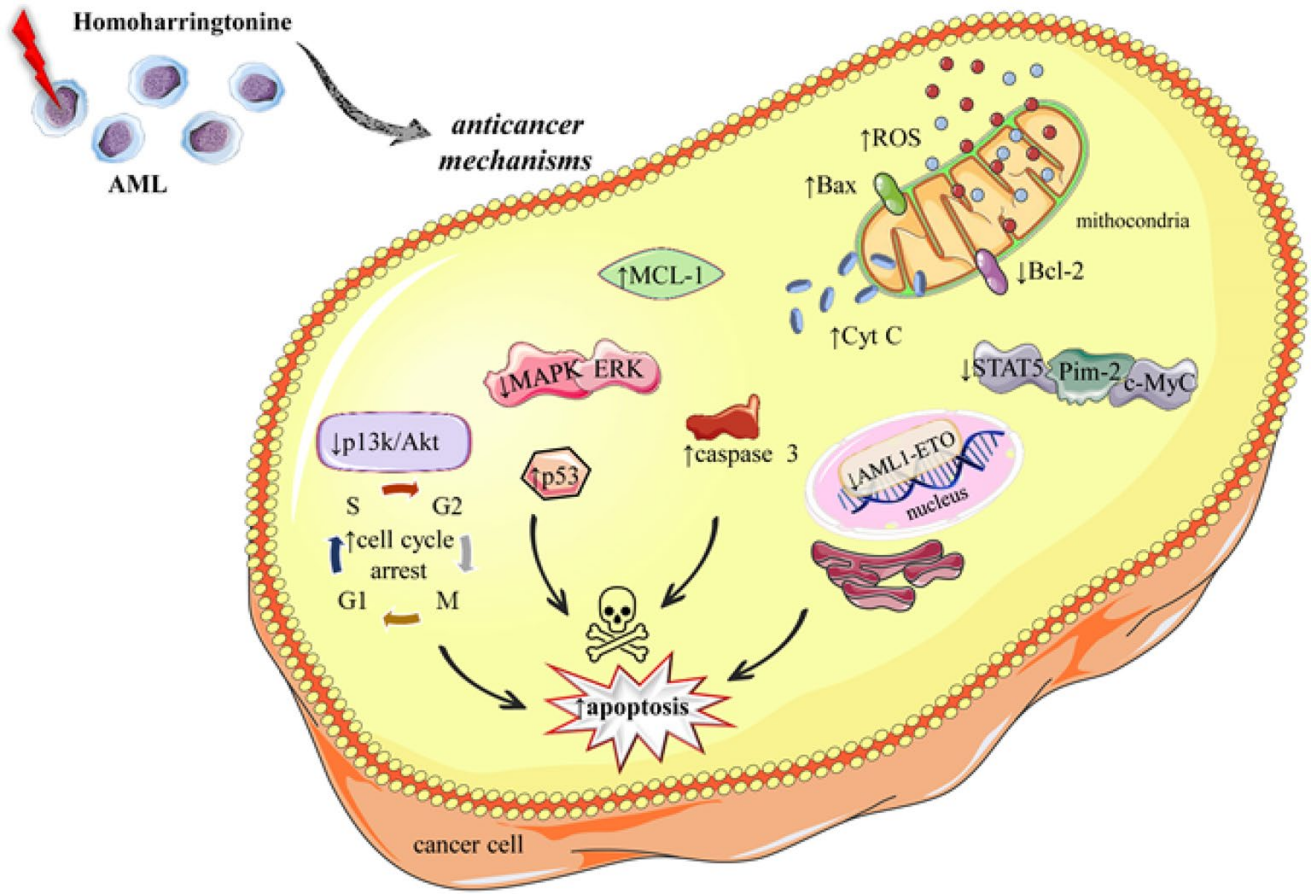
Illustrative figure regarding multifaceted mechanisms by which HHT exerts its antileukemic effects in AML treatment. HHT influences various cellular pathways leading to increased Reactive Oxygen Species (ROS), upregulation of Bax, and downregulation of Bcl-2 within the mitochondria, contributing to cytochrome c (Cyt C) release and apoptosis induction. It also inhibits the STAT5, Pim-2, and c-Myc oncoproteins, disrupts cell cycle progression by arresting at the G1/S and G2/M checkpoints through PI3K/Akt pathway modulation, and enhances the tumor suppressor p53 activity. Furthermore, HHT reduces MCL-1 levels, thereby facilitating apoptosis. These actions culminate in the activation of caspase 3, a pivotal executioner of apoptosis, leading to the death of cancer cells. AML: Acute Myeloid Leukemia; Bax: Bcl-2-associated X Protein; Bcl-2: B-cell lymphoma 2; Cyt C: Cytochrome c; ERK: Extracellular Signal-Regulated Kinase; HHT: Homoharringtonine; MAPK: Mitogen-Activated Protein Kinase; MCL-1: Myeloid Cell Leukemia 1; PI3K/Akt: Phosphoinositide 3-Kinase/Protein Kinase B; ROS: Reactive Oxygen Species; STAT5: Signal Transducer and Activator of Transcription 5

Figure 5 summarizes the anticancer mechanisms of HHT in the treatment of AML.

### Clinical studies

Till date, several studies have been performed focusing the clinical trials of HHT or OM against AML (Table 3). The HHT, venetoclax, and azacitidine combination (HVA) demonstrated improved treatment responses in patients with refractory/relapsed acute myeloid leukemia (R/R-AML), particularly in those who underwent allogeneic hematopoietic stem cell transplantation (allo-HSCT), and was well tolerated [79]. In another study, a single-centre phase II study with 46 patients (R/R-AML) was conducted by Yu et al. [80] to determine the effect of HAA. Eighty percent of patients achieved CR with an overall survival (OS) rate of 42%. Recently, researchers conducted a national, multicenter, randomized, double-blind, phase III clinical trial to assess the effectiveness of the HAA regimen in newly diagnosed AML patients. The trial's results indicated that the HAA regimen, an HHT-based triple drug combination, significantly increased the complete remission rate and extended overall survival, making it a promising treatment choice for young patients with newly diagnosed AML [81, 82].

**Table 3:** Preclinical and clinical studies regarding the efficacy of HHT and OM in AML: mechanisms and synergistic interactions

Types of studies			
Preclinical studies using cell lines (in vitro) or animal model (in vivo)			
Experimental model	Mechanisms	Results	References
HL-60 cells (In vitro)	↓ Protein synthesis Synergy with l-β-d-arabinofuranosylcytosine	↓ Protein synthesis Synergistic effect with DNA synthesis inhibitor	[67]
AML cells (In vitro)	↑ Bax	↑ Apoptosis No cross-resistance with DNR and cytarabine	[68]
HL60 HL60/MRP cells (In vitro)	↑MCL-1 turnover ↑Mitochondrial disruption ↑Caspases	↑ Apoptosis through mitochondrial pathway	[69]
AML cell lines Xenograft mice (In vitro/In vivo)	↓ MMP ↓ Mcl-1 ↓ c-KIT levels ↑Caspase-3	↑ Apoptosis Prolonged t(8;21) leukemia mouse survival ↑Synergy with oridonin	[61]
AML cells (In vitro)	↓ PI3K/AKT ↓WNT/β‐catenin signalling	↑ Apoptosis ↑Caspase-3 mediated cleavage of AML1–ETO oncoprotein ↑synergy with aclarubicin	[71, 72]
AML cell lines Primary AML cells (In vitro)	↑ ROS synthesis Restriction of antioxidant defence	Synergistic cytotoxicity with etoposide ↑ Apoptosis	[73]
FLT3–ITD positive cell lines Primary leukemia cells (In vitro)	↓STAT5/Pim-2/C-Myc ↑ Cell cycle arrest	↑ Apoptosis Alone or in combination: ↑ Sensitivity to chemotherapeuthic drugs	[74, 83]
AML cells Xenograft mice (In vitro/In vivo)	: ↓ MMP, ↓ Mcl-1, ↓ PI3K–AKT/MAPK/ERK pathways	↓ AML progression Prolonged survival in mice Synergistic effect with venetoclax	[70]

Clinical studies			
Cancer type	Study design	Primary outcomes	References
R/R-AML	Combination of HHT with venetoclax and azacitidine (HVA) Focus on patients with allo-HSCT	Better treatment response Well tolerated	[79]
R/R-AML	Single-center Phase II study with 46 patients HHT with aclarubicin and cytarabine (HAA)	80% achieved Complete Remission (CR) 42% OS rate	[80]
De Novo AML	National, multicenter, randomized, double-blinded, prospective Phase III clinical trial; HAA regimen	High CR rate Prolonged OS Suggested as a treatment option for young and newly diagnosed patients	[56, 81]

AML: Acute Myeloid Leukemia; Bax: Bcl-2-associated X protein; CR: Complete Remission; DNR: Daunorubicin; FLT3–ITD: FMS-like tyrosine kinase 3–internal tandem duplication; HHT: Homoharringtonine; HVA: HHT, Venetoclax, and Azacitidine combination; HAA: HHT, Aclarubicin, and Cytarabine combination; MCL-1: Myeloid cell leukemia 1; MMP: Mitochondrial membrane potential; OM: Omacetaxine Mepesuccinate; OS: Overall Survival; PI3K–AKT: Phosphoinositide 3-kinase–Protein kinase B; R/R-AML: Refractory/Relapsed Acute Myeloid Leukemia; ROS: Reactive oxygen species; STAT5/Pim-2/C-Myc: Signal transducer and activator of transcription 5 / Proviral integration site for Moloney murine leukemia virus-2/Cellular Myelocytomatosis; allo-HSCT: Allogeneic Hematopoietic Stem Cell Transplantation

### The impact of HHT on CML treatment

#### Disease overview

CML, also known as chronic myeloid leukemia, is a slow progressing hematopoetic disorder featured by malignant expansion of the pluripotent bone marrow stem cells and their accumulation in the blood (Table 4). The disease is slightly more common in men and has a reported incidence of 1–2 cases per 100,000, accounting for 15–20% of newly diagnosed leukemia cases in adults [84]. The clinical manifestations of this disease include leucocytosis (high white blood cell count), basophilia (elevated absolute basophil count) and splenomegaly (an enlarged spleen) [8]. It has been hypothesized that various factors such as smoking, exposure to high-dose radiation, pesticides, obesity, weight gain in adulthood, benzene and solvents, hair dye, and extremely low frequency electromagnetic fields, may contribute as potential risk factors [85]. The overall median age at diagnosis is 53 years; however, the disorder is manifested in all age groups, including children [84]. The characteristic cytogenetic feature of Chronic Myeloid Leukemia (CML) is the fusion of the Abelson murine leukemia (ABL1) gene on chromosome 9 with the breakpoint cluster region (BCR) gene on chromosome 22, known as translocation t(9;22)(q34;q11). This leads to the formation of the Philadelphia (Ph) chromosome, resulting in a BCR–ABL fusion oncogene and the production of a BCR–ABL fusion oncoprotein. The BCR–ABL protein is a constantly active tyrosine kinase that activates several intracellular signaling pathways, including RAS, RAF, JUN Kinase, MYC, and STAT. This activation contributes to genomic instability, abnormal cell proliferation, and the expansion of CML cell populations [86]. Starting in the 1980s, interferon (IFN)-α was the primary treatment for Chronic Myeloid Leukemia (CML), offering a reduction in disease burden and a modest improvement in survival compared to other treatments. However, the limited effectiveness of IFN-α and its frequent severe side effects led to its replacement in 2001 by targeted therapies using BCR–ABL tyrosine kinase inhibitors (TKIs) [87]. In 2002, FDA approved TKI namely imatinib mesylate (a small molecule Abl kinase inhibitor) to cure CML that has revolutionized the treatment. Imatinib directly binds to *BCR–ABL* kinase domain, prevents transfer of a phosphate group to tyrosine residue, hinders subsequent activation of phosphorylated protein and induces leukemic cell apoptosis [88]. Resistance to imatinib, a common issue in patients, often arises due to point mutations, such as T315I, Y253H, and F317L. These mutations lead to amino acid substitutions in the ABL kinase domain of the BCR–ABL protein, which disrupt the binding of imatinib [89]. In cases where resistance to imatinib occurs, second-generation tyrosine kinase inhibitors (TKIs) such as dasatinib (Sprycel; Bristol-Myers Squibb), nilotinib (Tasigna; Novartis Pharmaceuticals), and bosutinib are typically used. If a patient shows treatment failure with a second TKI, another TKI may be administered, or they might undergo allogeneic hematopoietic stem cell transplantation (allo-HSCT). Despite the significant successes of these treatments, resistance in CML patients has been observed, particularly in those with a mutation leading to the substitution of threonine with isoleucine at position 315 (T315I) in the BCR–ABL1 kinase domain. The T315I mutation, occurring in approximately 2–20% of CML cases, is notably problematic as it confers resistance to all currently available TKIs and represents one of the most challenging aspects in the treatment of CML [90]. Ponatinib, a third-generation tyrosine kinase inhibitor (TKI), is currently the only TKI recognized as effective in treating patients with the T315I mutation. [91, 92]. However, the responses observed with Ponatinib are typically short-lived, and the rates of discontinuation due to various reasons are high. Therefore, there is an urgent need for new therapeutic options for patients who have experienced treatment failure after trying multiple TKIs [91, 93]. In this scenario, HHT could be considered as an effective alternative as the drug is not a TKI and thus is independent on BCR–ABL binding for its therapeutic effects [91].

**Table 4:** Summarized data of acute myeloid leukemia (AML) and chronic myelogenous leukemia (CML): disease characteristics and treatment modalities

Disease characteristics	AML	CML	References
Nature of disease	Originates from leukemia stem cells; marked by increased myeloid cells in bone marrow	Slow-progressing expansion of pluripotent bone marrow stem cells	[57]; [84]
Prevalence	Most common acute leukemia in adults; 70% of acute leukemias	Accounts for 15–20% of adult leukemia cases	[58]; [84]
Clinical features	Hematopoietic insufficiency with/without leukocytosis	Leucocytosis, basophilia, splenomegaly	[57]; [8]
Risk factors	Genomic heterogeneity, selective treatment pressure	Smoking, radiation, pesticides, obesity, solvents	[59]; [85]
Age of onset	Higher prevalence in patients ≤ 60 years	Median age 53 years, affects all age groups	[62]; [84]
Cytogenetic markers	t(8;21) translocation, FLT3 mutations	Philadelphia chromosome from BCR–ABL gene fusion	[60, 62, 86]
Initial treatment approaches	Induction chemotherapy, “3 + 7” regimen, allo-HSCT	IFN-α, BCR–ABL TKIs (e.g., imatinib)	[64, 65, 87, 88]
Challenges and resistance	Disease relapse, drug resistance	Resistance via BCR–ABL kinase domain mutations	[65, 89]
Advanced treatment options	Exploration of novel, less toxic strategies	Ponatinib for T315I mutation; exploration of new therapies	[65, 90, 93]
Role of HHT	Effective in enhancing apoptosis, reducing proliferation	Alternative therapy for BCR–ABL TKI-resistant cases	[67, 68, 91]

AML: Acute Myeloid Leukemia; BCR–ABL: Breakpoint Cluster Region–Abelson; CML: Chronic Myelogenous Leukemia; FLT3: Fms-related tyrosine kinase 3; HHT: Homoharringtonine; IFN-α: Interferon Alpha; TKI: Tyrosine Kinase Inhibitor

### Pre-clinical studies

Initial studies evaluating the effect of HHT in CML were predominantly performed in the 1990s [94]. Several in vitro studies have been conducted till date to understand the efficacy of HHT on CML cells both along or in combination with IFN-α and/or ara-C. Visani et al. [95] described that HHT could exert more cytotoxicity against CP CML cells in comparison with normal bone marrow. OM has demonstrated preclinical activity in ponatinib-resistant BCR–ABL^+^ cell lines with Y253H, E255K, and T315I mutations [96]. In addition, a study employing primitive CD34^+^CD38^−^ leukemia initiating cells (LICs) from CML patients showed that OM can effectively kill BCR–ABL^+^ LICs in vitro [55].

**Fig. 6:**
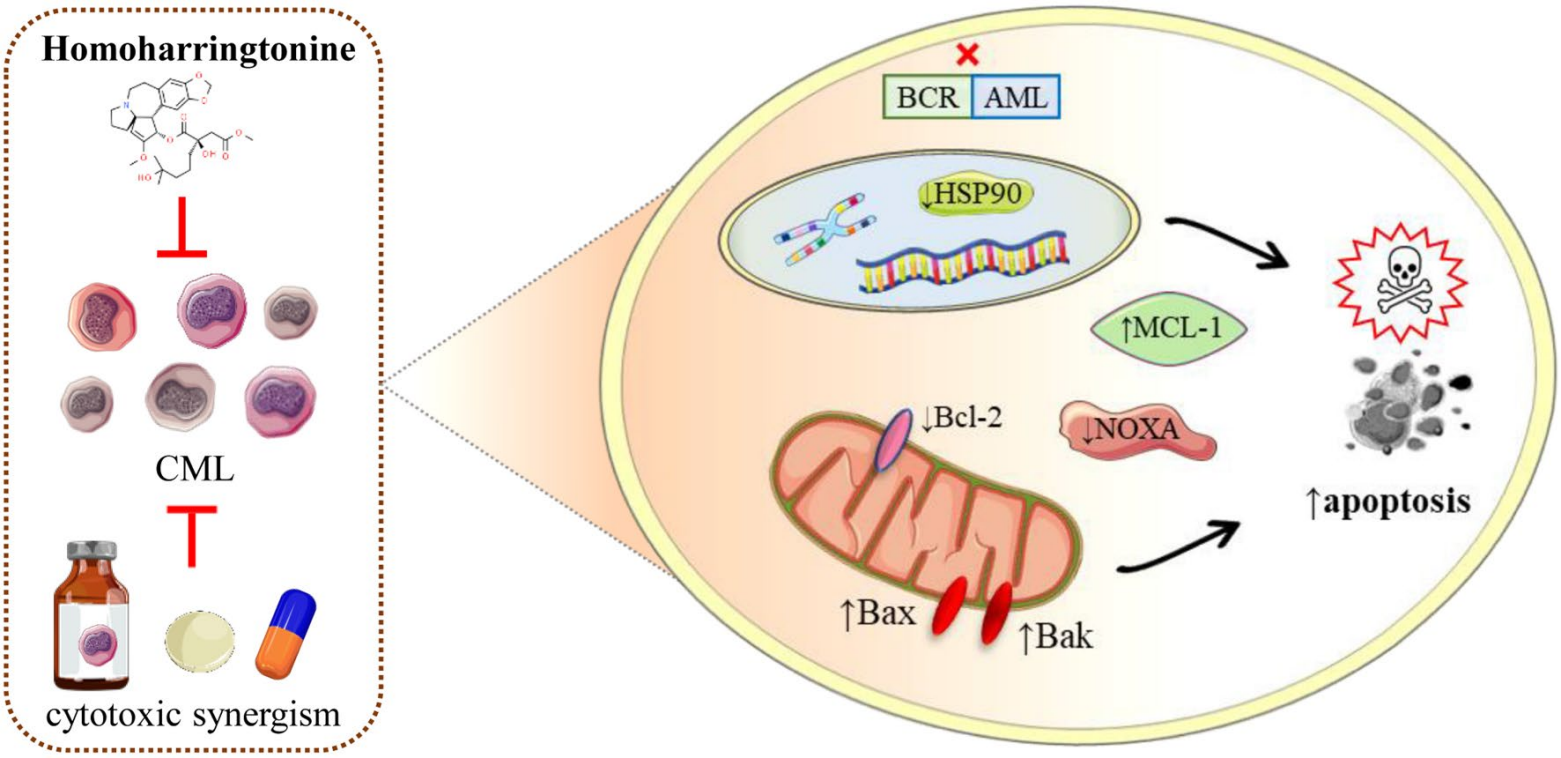
Impact of HHT on apoptosis induction in CML. HHT disrupts the BCR–ABL oncogenic signaling in CML, leading to the inhibition of the Heat Shock Protein 90 (HSP90) chaperone function. This disruption contributes to the downregulation of Myeloid Cell Leukemia 1 (MCL-1) and upregulation of NOXA, both of which are key regulators of apoptosis. Furthermore, the figure depicts the increase in Bcl-2-associated X Protein (Bax) and the activation of BAK, triggering the apoptosis cascade. The combination of HHT with other cytotoxic agents is shown to result in a synergistic effect, enhancing the apoptotic response in CML cells. Abbreviations and symbols: HHT: Homoharringtonine; CML: Chronic Myeloid Leukemia; BCR–ABL: Breakpoint Cluster Region–Abelson; HSP90: Heat Shock Protein 90; MCL-1: Myeloid Cell Leukemia 1; NOXA: Latin for “damage,” a pro-apoptotic Bcl-2 family member; Bax: Bcl-2-associated X Protein; BAK: Bcl-2 homologous antagonist/killer; ↑: Increase/upregulation; ↓: Decrease/downregulation; X: Inhibition/blockage.

Chen et al. [97] also conferred that OM effectively targets BCR–ABL^+^ LICs in animal models of Ph^+^ CML also and deliberates a substantial survival benefit for leukemic mice. They postulated three underlying potent pathways:

- By directly suppressing the expression of BCR–ABL.
- By decreasing the level of heat shock protein-90 (HSP90), a stabilizing protein of BCR–ABL, which may lead to the degradation of BCR–ABL.
- By diminishing expression of the short-lived antiapoptotic Bcl-2 family protein MCL-1 which is capable of stimulating cell survival (Fig. 6).

In another research, HHT has been shown to possess a synergistic or additive effect with imatinib against a BCR–ABL^+^ cell line with an imatinib-resistant E255K mutation and cells from patients with CML in blastic phase (BP) [98, 99]. Nguyen et al. indicated that HHT and bortezomib an inhibitor of 20S proteasome) cooperatively can kill diffuse large B-cell lymphoma (DLBCL) and mantle cell lymphoma cells through a process involving MCL-1 down-regulation, NOXA up-regulation and Bak activation [100]. The combination of bortezomib and HHT also exerted synergistic anti-proliferative activity against K562 cells and induced apoptosis via suppression of Bcl-2 protein and up-regulation of Bax protein [101]. All these scientific findings along with traditional use encouraged for further clinical trials of HHT. 

### Clinical studies

One of the early studies on clinical trial of HHT in CML patients was reported by O’Brien et al. in 1995 [102]. A continuous infusion of HHT at a daily dose of 2.5 mg/m^2^ was considered for 2 weeks for remission induction and for 1 week every month for maintenance. Results enumerated 72% complete hematologic response (CHR) and 31% cytogenetic (CG) response rate [complete CG response (Ph 0%) in 15% of patients]. Later on, the same research team administered six courses of HHT to 90 CML patients at early CP (< 1 year from diagnosis); they were later switched to maintenance therapy with IFN-α. Analysis showed that both the CHR and CG rates in patients treated with natural HHT and INF-α were markedly higher than those recorded in historical control patients receiving 6 months of only INF-α therapy [103]. O’Brien et al. [104] also reported CG responses in 66% of 47 CML patients in CP using HHT at a dose of 2.5 mg/m^2^ per day by constant infusion over 5 days in combination with IFN-α (intended dose 5 MU/m^2^ per day).

The combination regimen of HHT and ara-C is also considered as effective and safe in patients with CML who have experienced treatment failure with IFN-α. In a clinical trial, 100 patients were subjected to 2.5 mg/m^2^ HHT daily for 5 days and 15 mg/m^2^ ara-C daily for 5 days every month. Interestingly, response rates were found to be identical with HHT together with ara-C versus HHT alone; but the survival rate was comparatively longer with the combination [105]. Recently, Stone et al. [8] performed a phase II trial of HHT (2.5 mg/m^2^ per day) plus cytarabine (7.5 mg/m^2^ per day), given together via continuous intravenous infusion for 7 days in previously untreated patients with Ph chromosome positive CP CML and the cycles were repeated every 28 days. Results showed that 36 of 44 patients (82%) achieved a complete hematologic remission, whereas a major cytogenetic response (MCyR) was achieved by 17%.

To understand the pharmacokinetics of HHT along with TKI, 13 patients with CML who accomplished a suboptimal response to imatinib alone had s.c. semisynthetic HHT at a dose of 1.25 mg/m^2^ twice daily for 1 day every 28 days supplemented with their ongoing imatinib therapy. Amongst them, 10 patients were evaluated, of them 5 had their BCR–ABL transcript levels decreased by more than 1−log [106]. The effectiveness of HHT after imatinib failure was evaluated by Quintás-Cardama et al. [107] by administrating an i.v. loading dose of HHT 2.5 mg/m^2^ for 1 day, followed by 1.25 mg/m^2^ s.c. (twice daily for 2 weeks every month). Amongst the five evaluable patients, three had CG response; while the BCR–ABL kinase domain mutations became undetectable in two patients. Triple therapy with natural HHT, INF-α, and cytarabine, followed by imatinib, resulted in an estimated 5-year survival rate of 88% [69]. Recently, a multi-center phase II trial has been performed encompassing 46 CML patients who had failed two/more prior TKIs. To understand the efficacy of OM, the drug was administered at the level of 1.25 mg/m^2^ subcutaneously twice daily for continuous 2 weeks every 28 days. People who tolerated them were subjected to OM at the same dose for 1 week every 28 days. Strikingly, 67% of hematologic responses were reported and MCyR was achieved in 22% of patients. The observed tolerability profile of OM was acceptable [91]. The same fashion of OM has been used in another phase II trial including 62 patients who had failed prior TKI therapy and harboured the T315I mutation. Results showed that 48 and 14 patients achieved CHR and MCyR, respectively. The therapy was well-tolerated but cytopenias (reduction in the number of mature blood cells) were frequent. This study highlighted the potential of OM as therapy for patients with CML carrying T315I mutated clones [89]. A triple combination regimen of IFN-α, ara-C, and HHT has also been assessed in patients with newly diagnosed Ph-positive CML. The treatment was followed by imatinib administration resulting an estimated 5-year survival rate of 88%. The outcome thus suggested that imatinib combination regimen might improve the prognosis in CML [108]. Overall, several Phase II studies tested HHT or OM either alone or in combination with other active agents for the treatment of patients with CML in different disease stages (Table 5).

**Table 5:** Preclinical and clinical studies regarding the efficacy of HHT and OM in CML: mechanisms and synergistic interactions

Types of studies			
Preclinical studies using cell lines (in vitro) or animal model (in vivo)			
Experimental model	Mechanisms	Results	References
CP CML cells (In vitro)	Cytotoxicity comparison with normal bone marrow	↑ Cytotoxicity against CP CML cells compared to normal bone marrow	[95]
Ponatinib-resistant BCR–ABL + cell lines (In vitro)	Activity against Y253H, E255K, and T315I mutations	OM demonstrated efficacy in ponatinib-resistant BCR–ABL + cell lines	[96]
CD34 + CD38–LICs from CML patients (In vitro)	Targeting leukemia initiating cells	OM effectively killed BCR−ABL + LICs	[55]
BCR–ABL + cell line with E255K mutation and CML BP cells (In vitro)	Synergistic/additive effect with imatinib	HHT showed synergistic effects with imatinib in resistant CML cell lines	[98, 99]
DLBCL and mantle cell lymphoma cells (In vitro)	Combination with bortezomib: ↓ MCL-1, ↑ NOXA, ↑ BAK	↑ Apoptosis anti-proliferative activity against K562 cells	[100, 101]
Ph + CML animal models (In vivo)	Targeting BCR–ABL + LICs; ↓ BCR–ABL; ↑ HSP-90 and MCL-1 levels	Substantial survival benefit in leukemic mice	[97]

Clinical studies			
Cancer Type	Study design	Primary outcomes	References
CML	Phase II: Continuous infusion of HHT for remission induction and maintenance	72% CHR, 31% CG response rate, 15% complete CG response	[102]
	Phase II: Six courses of HHT followed by IFN-α maintenance in 90 early CP CML patients	Higher CHR and CG rates compared to IFN-α alone	[103]
	Phase II: HHT with IFN-α in 47 CP CML patients	66% CG responses	[104]
	Phase II: HHT and ara-C combination in 100 CML patients failing IFN-α therapy	Similar response rates with HHT and ara-C vs. HHT alone; longer survival with combination	[105]
	Phase II: HHT plus cytarabine in 44 untreated Ph chromosome positive CP CML patients	82% achieved hematologic remission; 17% MCyR	[8]
	Phase II: HHT with ongoing imatinib therapy in 13 patients with suboptimal response to imatinib	50% decrease in BCR–ABL transcript levels	[106]
	Phase II: HHT after imatinib failure in 5 evaluable CML patients	CG response in 60%; undetectable BCR−ABL mutations in 40%	[107]
	Phase II: Triple therapy with HHT, INF-α, and cytarabine, followed by imatinib	Estimated 5-year survival rate of 88%	[69]
	Phase II: OM in 46 CML patients failing two or more prior TKIs	67% hematologic responses; 22% MCyR	[91]
	Phase II: OM in 62 CML patients with T315I mutation	CHR in 48 patients; MCyR in 14 patients	[89]
	Phase II: Triple combination of IFN-α, ara-C, and HHT, followed by imatinib	Improved prognosis with an estimated 5-year survival rate of 88%	[108]

BAK: Bcl-2 homologous antagonist/killer; BCR–ABL: Breakpoint Cluster Region–Abelson; CML: Chronic Myeloid Leukemia; CP: Chronic Phase; DLBCL: Diffuse Large B-cell Lymphoma; HHT: Homoharringtonine; HSP-90: Heat Shock Protein-90; LICs: Leukemia Initiating Cells; MCL-1: Myeloid cell leukemia 1; NOXA: Phorbol-12-myristate-13-acetate-induced protein 1; OM: Omacetaxine Mepesuccinate; Ph + : Philadelphia chromosome positive; BAK: Bcl-2 homologous antagonist/killer; BCR–ABL: Breakpoint Cluster Region–Abelson; BP: Blastic Phase; CHR: Complete Hematologic Response; CG: Cytogenetic; CML: Chronic Myeloid Leukemia; CP: Chronic Phase; DLBCL: Diffuse Large B-cell Lymphoma; HHT: Homoharringtonine; HSP-90: Heat Shock Protein-90; IFN-α: Interferon Alpha; LICs: Leukemia Initiating Cells; MCL-1: Myeloid cell leukemia 1; MCyR: Major Cytogenetic Response; NOXA: Phorbol-12-myristate-13-acetate-induced protein 1; OM: Omacetaxine Mepesuccinate; Ph + : Philadelphia chromosome positive

Today, HHT has been proved to be significantly active as salvage therapy for patients with CML after failure on IFN-α therapy. However, the noteworthy success of imatinib mesylate in this context shadowed potent activity of HHT. The therapeutic effect of OM in imatinib-resistant CML has again established this medication for the second time as a valuable option to manage the disease [12].

### Efficacy of HHT in other hematological malignancies

In addition to its application in the treatment of Chronic Myelogenous Leukemia (CML) and Acute Myeloid Leukemia (AML), Omacetaxine Mepesuccinate (OM) is also utilized in managing other blood malignancies. These include Myelodysplastic Syndromes (MDS), Chronic Lymphocytic Leukemia (CLL), B-cell Acute Lymphoblastic Leukemia (B-ALL), and Multiple Myeloma (MM).

Chen et al. [109] showed that HHT can induce apoptosis in CLL cells at nanomolar levels. The activity was mediated through protein synthesis inhibition, decrease in Mcl-1 concentration, loss of ΔΨm, PARP cleavage and proteasome degradation. Furthermore, HHT induced synergistic cell killing with SNS-032 (a transcription inhibitor) suggesting clinical development of the alkaloid in CLL as a single entity or in combination. In another research, Chen et al. [97] showed that OM can inhibit the proliferation of B-ALL stem cells. Further investigation described that all B-ALL mice treated with OM survived in contrast to all placebo-treated recipients conferring potent activity of OM.

Jin et al. demonstrated that HHT can effectively suppress the proliferation of HMC-1.2, HMC-1.1, and P815 mastocytoma cell line derived from a mouse. This inhibition is mediated through the decreased expression and phosphorylation of receptor tyrosine kinase KIT, along with the inhibition of phosphorylation in KIT-dependent downstream signaling molecules, including Akt, signal transducer and activator of transcription (STAT) 3 and 5, and extracellular signal-regulated kinase (ERK) 1/2. In addition, HHT significantly prolonged the survival of mice with aggressive systemic mastocytosis or mast cell leukemia, indicating its potential effectiveness in treating systemic mastocytosis (SM) [110].

Essential thrombocythemia (ET) is a myeloproliferative neoplasm, a type of blood malignancy characterized by the overproduction of platelets in the bone marrow [111]; this disorder leads to an increased risk of blood clot formation; often associated with genetic mutations like JAK2, ET may present with symptoms related to clotting or may be asymptomatic [111]. Treatment focuses on managing the risk of thrombosis and alleviating symptoms. In a clinical study, patients who are intolerant to IFN-α therapy or hydroxycarbamide were treated with a dose of 1.5 mg/m^2^ of HHT every month. After six courses of HHT therapy, the hematological response rate in essential thrombocythemia was observed to be 72.2% (13 out of 18 patients), and the remission rates for symptomatic splenomegaly and constitutional symptoms were 71.4% and 66.7%, respectively. However, the limited efficacy of lower doses of HHT alone was not long-lasting in cases of severe essential thrombocythemia. Therefore, HHT has been considered a second-line drug for treating essential thrombocythemia, particularly following hydroxycarbamide or IFN-α therapy [112].

### Effects of HHT on other types of cancers

#### Breast cancer

Breast cancer is a malignancy that develops in breast tissue, it often originates in the ducts or glands and can spread to other body parts and treatment typically includes surgery, radiation, and medication [113, 114]. Recent reports indicate that HHT can be effective in the treatment of solid tumors, particularly in breast cancer [69]. For instance, Yakhni et al. [115] reported that HHT at the levels of 20–100 ng/mL could inhibit the proliferation of MDA-MB-157, MDA-MB-468, CAL-51 and MDA-MB-231 by more than 80% within 2–3 days resulting low IC_50_ (half-maximal inhibitory concentration) values (Table 6). Further analysis portrayed in vivo growth inhibitory effects of HHT against two different models of aggressive TNBC following administration of a 7-day regimen. Recently, Wang et al. [11] demonstrated that HHT inhibited human breast cancer cell growth and proliferation in vivo and in vitro. Besides, HHT can also induce breast cancer cell apoptosis by regulated the expression of apoptosis-related protein, such as Bax/Bcl-2, Caspase3/Caspase9 and PARP. HHT consistently showed the most cytotoxicity towards an additional six TNBC cell lines (BT549, HCC1395, HCC38, Hs578T, MDA-MB-157, MDA-MB-436), and several luminal A breast cancer cell lines (HCC1428, MCF7, T47D, ZR-75-1). A strong synergy was observed using the combination of HHT and paclitaxel, with high cytotoxicity towards TNBC cells at lower concentrations than when each was used separately [18]. Isoharringtonine (IHT), a natural analogue of HHT, was found to have functional properties of decreasing the proliferation and migration of breast cancer stem cells via inhibition of the STAT3/Nanog pathway as well [116].

**Table 6:** Effectiveness of HHT in diverse cancer types

Cancer type	Model	Mechanisms	Results	References
Breast cancer (TNBC)	In vitro MDA-MB-157 MDA-MB-468 CAL-51 MDA-MB-231	↑ Bax/Bcl-2, ↑ Caspase 3, − 9 ↑ PARP ↓ STAT3/Nanog	↓ Proliferation ↑ Apoptosis synergy with paclitaxel	[18, 69, 115, 116, 120]
Lung cancer	In vitro A549 H1975 A549B/VP29	↓ TMEM16A, ↓ MAPK pathway ↓MEK1/2, ↓ERK1/2 ↓ IL-6/JAK/STAT3	↓ Tumor cells growth ↑ Cell cycle arrest ↑ Apoptosis	[17, 117–119]
Hepatocellular carcinoma	In vitro HepG2, Hep3B SMMC-7721 Bel-7402, Bel-7404 In vivo Mice	↑ Cell cycle arrest at S phase ↑ Hippo pathway ↑ EphB4/β-catenin	↓ Tumor growth ↑ Apoptosis ↓ Colony formation ↓ Cell invasion/migration	[51, 120, 121]
Colon cancer	In vitro LoVo SW480 HCT116 HCTl16/VP48	↓ EphB4, ↓ p-MEK ↓ p-ERK1/2 ↓ Wnt/β-catenin ↑ Caspases ↓ Bcl-2/Mcl-1, ↑ Bax/Bad	↓ Cell viability ↓ Colony formation ↑ Apoptosis ↑ Sensitivity in drug-resistant cell lines	[119, 123–126]

Bax: Bcl-2-associated X protein, Bcl-2: B-cell lymphoma 2, β-catenin: Protein in Wnt signaling, Caspase: Cysteine-aspartic proteases, EphB4: Erythropoietin-producing hepatocellular receptor B4, ERK1/2: Extracellular signal-regulated kinases 1 and 2, Hippo: Signaling pathway, IL-6: Interleukin 6, JAK: Janus kinase, MAPK: Mitogen-activated protein kinase, Mcl-1: Myeloid cell leukemia 1, MEK1/2: Mitogen-activated protein kinase kinase 1 and 2, Nanog: Stem cell proliferation protein, PARP: Poly (ADP-ribose) polymerase, p-ERK1/2: Phosphorylated ERK1/2, p-MEK: Phosphorylated MEK, STAT3: Signal transducer and activator of transcription 3, TMEM16A: Transmembrane protein 16A, TNBC: Triple-Negative Breast Cancer, Wnt/β-catenin: Cell proliferation and differentiation pathway.↑:increase, ↓: decrease

### Lung cancer

Several researchers have validated HHT as a potent drug for lung cancer owing to better safety and low cost. Guo et al. [17] showed that HHT can bind to K697 residues on TMEM16A (a protein which is particularly expressed in lung cancer tissues) blocking the ion channel activity. In addition, HHT down-regulated expression of TMEM16A protein causing de-phosphorylation of MEK1/2 and ERK1/2 in MAPK pathway that in turn resulted cell cycle halt and apoptosis induction. Further analysis at animal model demonstrated that HHT did not reduce body weight of mice verifying biological safety of HHT. It has been demonstrated that HHT can effectively inhibit the proliferation, viability, and soft-agar colony formation of Gefitinib-resistant non-small cell lung cancer (NSCLC) cells, specifically the A549 and H1975 lines. Further investigations revealed significant disruptions in mitochondrial membrane potential (MMP), fluctuations in Ca2 + levels, release of cytochrome c, and activation of caspase 9 and 3, along with the cleavage of PARP levels, thereby indicating the activation of the intrinsic apoptotic pathway. In addition, HHT significantly reduced tumor growth in vivo within a 3-week treatment period. Further molecular analysis indicated that HHT's anti-tumor effects are mediated through the inhibition of the IL-6/JAK/STAT3 signaling pathway, leading to the induction of cell apoptosis [117]. Similar to that, significant reduction in expression of oncogenic and tumor suppressor proteins were recorded when HHT was challenged against NSCLC cell lines in vitro and in vivo [118]. Moreover, etoposide, teniposide and vinblastine-resistant human lung cancer cells (A549B/VP29) exhibited sensitivity to HHT suggesting potent use of HHT in treating tumors with this type of drug resistance [119].

### Hepatocellular carcinoma

HHT could significantly inhibit hepatocellular carcinoma (HCC) cell proliferation by suppressing colony formation, repressing cell invasion and migration, inducing cell cycle arrest at S phase and promoting apoptosis mediated through Hippo pathway. In vivo analysis further endorsed tumor inhibitory effect of HHT [120]. In a separate study, HHT was depicted to restrain HCC proliferation and migration through an EphB4/β-catenin dependent manner [121]. The antitumor and antifibrotic effects of HHT were also validated by subcutaneous xenograft tumor and CCL_4_ induced liver fibrosis models. Using Library of Integrated Cellular Signatures approach, HHT was also identified as liver cancer preventive candidate [122].

### Colon cancer

The impact of HHT on colorectal cancer and its potential mechanism of action have been subjects of recent investigation. Research has shown that HHT significantly inhibits the viability and colony formation of LoVo cells, inducing cell cycle arrest at the S phase and depolarization of mitochondrial membrane potential (ΔΨm), while not affecting normal cells, such as HEK293. Mechanistic studies revealed that HHT suppresses EphB4 expression in LoVo cells by inhibiting phosphorylated MEK (p-MEK) and phosphorylated ERK1/2 (p-ERK1/2), and by activating phosphorylated P38 (p-P38). In addition, HHT reduces the expression of Bcl-2 and Mcl-1, increases the levels of Bax and Bad, and activates caspase 3, 7, and 9 [123].

Similar to that, Qu et al. [124] reported that HHT inhibited LoVo and SW480 cell proliferation, cell cycle progression, colony formation, migration and invasion, and promoted apoptosis mediated through inactivation of PI3K/AKT/mTOR signaling. Moreover, HHT repressed CRC tumor growth in nude mice. In another study, HHT induced apoptosis in HCT116 cells through suppressing β-catenin effect and inhibiting the protein expression levels of Wnt/β-catenin signaling key factors as well as short-lived proteins such as c-Myc [125]. HHT has also been effective in suppressing colorectal cancer in vitro and in vivo by liaising with anti-TNF-related apoptosis-inducing ligand (TRAIL) antibody [126]. Moreover, etoposide, vincristine and vinblastine-resistant human colon cancer cells (HCTl16/VP48) exhibited sensitivity to HHT [119].

**Fig. 7:**
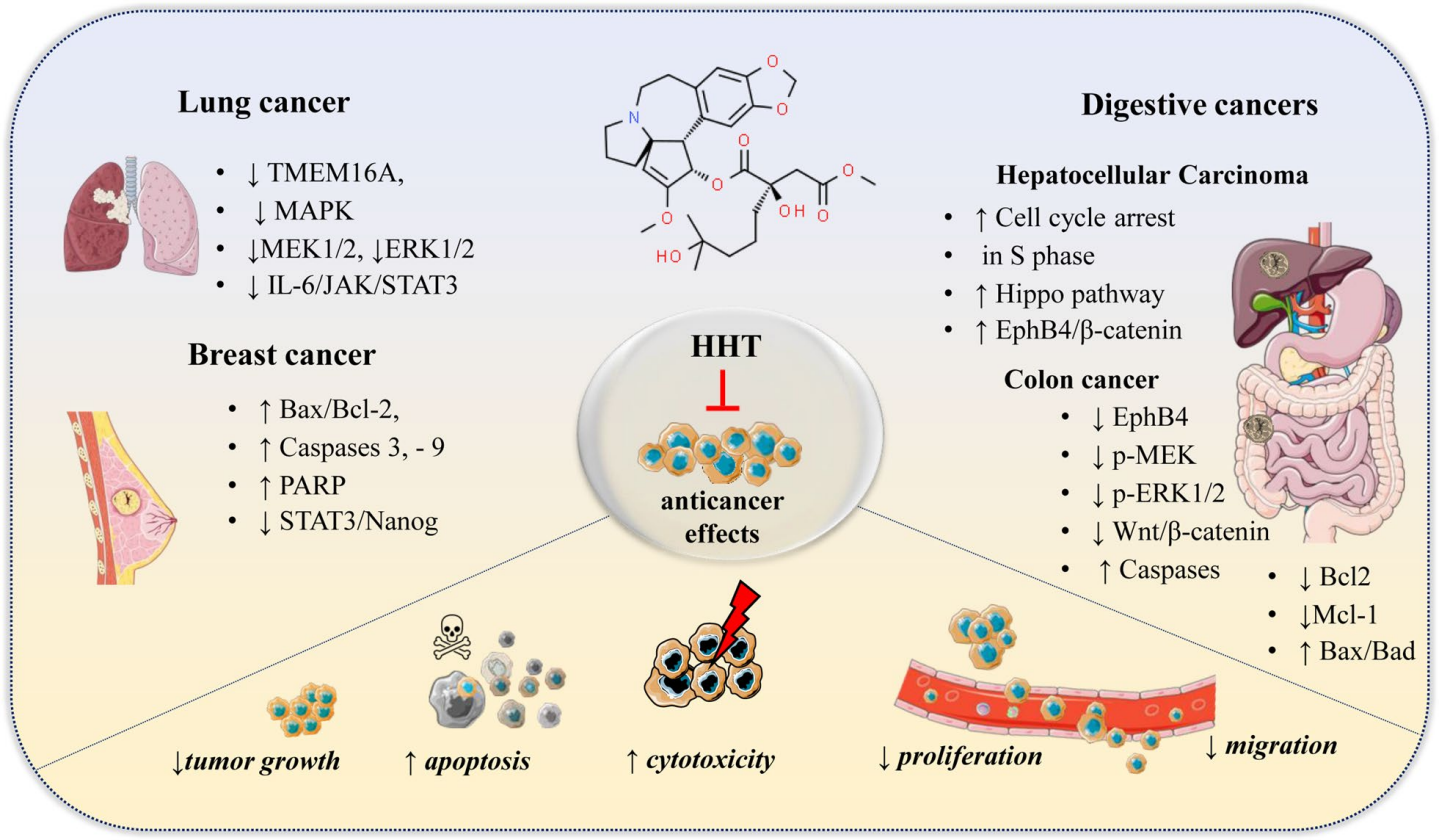
Anticancer mechanisms of HHT in various cancer types: inhibition of proliferation, induction of apoptosis, and disruption of cell signaling pathways across multiple cancer types. Akt—Protein kinase B; Bax—Bcl-2-associated X; Bcl-2—B-cell lymphoma 2; Caspases—cysteine–aspartic proteases; ERK—extracellular signal-regulated kinases; Hippo—signaling pathway; IL-6—Interleukin 6; JAK—Janus kinase; MAPK—mitogen-activated protein kinase; MEK—mitogen-activated protein kinase kinase; Nanog—stem cell proliferation protein; PARP—poly-(ADP-ribose) polymerase; STAT3—signal transducer and activator of transcription 3; TMEM16A—transmembrane protein. Symbols: ↑—increase/upregulation; ↓—decrease/downregulation

Table 6 and Fig. 7 present a summary of the effectiveness of HHT across various cancer types, including TNBC, lung cancer, hepatocellular carcinoma, and colon cancer, highlighting specific molecular mechanisms and observed results.

## Other pharmacological applications of HHT

### Anti-inflammatory activity

Inflammatory diseases encompass a range of conditions marked by immune-mediated inflammation in the body, often resulting in pain, swelling, and reduced function [127] Natural products with anti-inflammatory properties have long been used as folk remedies for conditions such as pain, arthritis, migraines, and fever [128]. The variety of non-steroidal anti-inflammatory drugs, as well as anti-inflammatory foods and products, have become increasingly intriguing following detailed studies of the underlying causes of inflammatory diseases [129]. Reports indicate that post-laminectomy, surgery-induced epidural fibrosis is often aggravating, with fibroblast proliferation being a key factor in its formation. Both in vitro and in vivo experiments have shown that HHT suppresses fibroblast proliferation and epidural fibrosis formation, while also inducing apoptosis. The expression of key endoplasmic reticulum (ER) stress marker proteins, such as glucose-regulated protein and C/EBP homologous protein, were observed to be positively altered. In summary, HHT may exert its effects through the ER stress signaling pathway, leading to the apoptosis of fibroblasts [20].

### Anti-parasitic activity

In many tropical developing countries, parasitic diseases continue to be a significant public health concern, impacting the lives of millions. There is growing interest in the potential of natural derivatives, which could contribute to the development of much-needed new antiparasitic drugs [130]. *Trypanosoma brucei*, the parasite responsible for the deadly sleeping sickness, commonly referred to as Chagas disease, has a limited number of available medications. Plant alkaloids, such as HHT, are increasingly recognized as potent trypanocidal agents. Research indicates that pretreatment with such alkaloids significantly inhibits the growth and protein biosynthesis of these parasites in both a concentration- and time-dependent manner. Moreover, HHT disrupts the inner mitochondrial membrane potential and arrests the normal cell cycle. However, it does not affect an essential component of the trypanosomes' redox system, the trypanothione reductase [131].

### Neuroprotective activity

Alzheimer's disease, commonly occurring in the elderly population, is a chronic and progressive neurodegenerative disorder responsible for age-related dementia and cognitive impairment [132]. Recently, pharmacologically active, nature-derived products with significant anti-neuroinflammatory activity have garnered considerable attention, positioning them as potential candidates for the treatment of Alzheimer’s disease [133]. Research on neuroprotective and anti-inflammatory phytochemicals, including alkaloids, steroidal saponins, terpenoids, glycosides, and phenolic derivatives, has shown promising efficacy in mitigating the effects of devastating neurodegeneration [134]. HHT is an alkaloid reported to have significant potential in slowing the progression of Alzheimer’s disease. In vivo experiments showed that injection of 2 mg/kg HHT for every alternative day into APP/PS1 mice for half-yearly resulted in significant alleviation of cognitive deficits [19]. Various studies demonstrated that neuroinflammation connected with accumulation of amyloid β peptide in the brain is a prime marker towards the pathology of neurodegenerative diseases, such as Alzheimer’s disease [135]. Treatment with HHT has significantly reduced the accumulation of both soluble and insoluble Aβ40 and Aβ42 peptides and alleviated the impairment of synaptic function in the hippocampus of Alzheimer's disease mouse models. The mechanism through which HHT mitigated neuroinflammation in the APP/PS1 mouse hippocampus involved increasing the expression of phosphorylated SOCS3 (Suppressor of Cytokine Signaling 3) and decreasing the activation of STAT3 (Signal Transducer and Activator of Transcription 3) [19].

### Antiallergic activity

The activation and degranulation of mast cell are the vital inflammatory component of the allergic process; during the allergic process variety of inflammatory mediators such as histamine, leukotrienes, several cytokines and platelet aggregating factor were released from mast cells [136, 137]. Currently medicinal plants are widely used for the treatment of every other disease which is connected with folk medicine of various parts of the globe [138, 139]. The exploration for novel anti-allergic agents were magnifying every day from the huge array of medicinal plant resources which is available to us [140]. Studies have shown that exposure to HHT suppressed allergic reactions and alleviated clinical symptoms of atopic dermatitis in a mouse model. This model was induced by anaphylaxis using 2,4-dinitrophenyl-human serum albumin (DNP–HSA) and 2,4-dinitrofluorobenzene (DNFB). HHT effectively reduced both passive systemic and cutaneous anaphylaxis, as well as the expression of cytokines like Th1/Th2. The results clearly indicate the potential of HHT as a candidate for the development of anti-allergy drugs, acting through the modulation of the NF-κB-miR-183-5p-BTG1 axis in rat basophilic cells [141].

### Cardioprotective effect in diabetes-induced ischemic heart disease

Diabetes, a chronic condition marked by elevated blood sugar levels, can lead to serious health complications, such as heart disease, kidney failure, and vision impairment; the global increase in diabetes is largely driven by the widespread prevalence of unhealthy lifestyles [142]. Ischemic heart disease (IHD), one of the prime causes for mortality in diabetic individuals [143]. Diabetes has remarkably elevated the death of ischemic heart disease by worsening cardiac injury caused by ischemia [144]. HHT selectively inhibited ribosomal function and induced apoptosis in cardiomyocytes in both normal diet (ND)-fed and high-fat diet (HFD)-induced diabetic mice. This was evidenced by the elevated expression of caspase 3 and an increase in TUNEL-positive cardiomyocytes. Consequently, HHT administration effectively negated the effects of nucleostemin overexpression on protein synthesis, as well as the cardioprotective effects of nucleostemin against ischemia/reperfusion (I/R) injury [145].

### Anti-viral activity

Viral infections play a crucial role in human diseases, and the recent surge in globalization and ease of travel has highlighted their prevention as a critical issue in protecting public health [3]. In spite of the advancement made in drug development and immunization, several viruses still abide without any efficacious immunization, and hence, significant antiviral therapies are needed [146]. Herbal medicines and purified nature derived compounds furnish a richest resource for new antiviral drug development [147]. HHT, an alkaloid ester derived from the genus *Cephalotaxus*, has been reported to exhibit potent antiviral activity. In vitro experiments demonstrated that a concentration of 10 ng/mL of HHT significantly inhibited the replication of recombinant varicella-zoster virus (VZV)–pOka luciferase. Furthermore, treating cells with the same dose effectively suppressed the lytic gene expression of varicella-zoster virus and showed potent antiviral efficacy against a clinical isolate of the virus [148]. The antiviral activity of HHT was evaluated against *Flavivirus*, using bovine viral diarrhea virus (BVDV) as a surrogate for hepatitis C virus (HCV). HHT demonstrated toxicity in embryonic bovine trachea cells and exhibited an inhibitory effect against bovine viral diarrhea virus. In addition, the alkaloid was capable of suppressing hepatitis B virus production at concentrations 10 to 100 times lower than those required to induce cell toxicity. Therefore, HHT could potentially be beneficial in combined therapy against hepatitis B virus. [149]. HHT displayed wide antiviral activity both in vitro as well as in vivo. It has entirely prevented the infections of vesicular stomatitis virus (VSV), porcine epidemic diarrhea virus (PEDV) and newcastle disease virus (NDV) at a dosage of 50, 500 and 100 nmol/L, respectively. The alkaloid ester was also capable of alleviating disease symptoms along with suppressing viral load at a concentration of 0.2 and 0.05 mg/kg in newcastle disease virus (NDV) and porcine epidemic diarrhoea virus (PEDV), respectively. The infection of echovirus 1 (EV1) [150], herpes simplex virus type 1 (HSV-1) and avian influenza virus (AIV) was moderately restricted by HHT, though it also bear potent anti-viral efficacy against various RNA viruses [13]. Porcine epidemic diarrhea virus (PEDV) belongs to the Coronaviridae family has resulted in huge economic losses in the swine husbandry industry. At a low dose of 150 nM, HHT decreased the virus titer in TCID50 by 30 and 3.5 fold, respectively, and the combinatorial treatment has further lowered the virus titer in TCID50 by 200 fold [151]. HHT remarkably restricted the wild-type zika virus (ZIKV) infection at the post-entry stage in cells of BHK-21 and human cell line A549 in a dose dependent manner at a concentration of 0.9 µmol/L [152]. FDA-approved HHT for the treatment of leukaemia as it effectively obstructs the elongation of protein elongation [153]. Hence reports claimed that HHT could efficiently repress the coronaviruses replication at lowest concentrations. In a recent in vivo experiments demonstrated that within 3 day SARS-CoV-2 has been perfectly cleared with 40 µg HHT by nasal dripping. Dogs were also used as an experimental model to validate the safety of HHT delivery by nebulisation. Similarly Ma et al. [154] also confirmed the safety of HHT nebulisation to combat the coronavirus disease 2019 (COVID-19) which is caused by SARS-CoV-2. Other reports also confirmed the promising therapeutic activity of HHT against the most dangerous virus SARS-CoV-2 (COVID-19) with an IC_50_ value of 2.10 μM [155, 156]. Cumulatively the studies indicate that HHT bear significant potency to be repurposed as a wide-spectrum antiviral drug. Table 7 summarizes other pharmacological properties of HHT.

**Table 7:** HHT biomedical applications: mechanisms and outcomes across various models

Pharmacological property	Model	Molecular Mechanisms	Results	References
Anti-inflammatory	In vitro In vivo	ER stress signaling pathway	↓ Fibroblast Proliferation ↑ Apoptosis ↓ Epidural fibrosis	[75]
Anti-parasitic	In vitro	Disruption of mitochondrial membrane potential, ↑ Cell cycle arrest	↓ Tumor growth ↓ Protein biosynthesis of *Trypanosoma brucei*, not affecting trypanothione reductase	[131]
Neuroprotective	In vivo APP/PS1 mice	↓ Neuroinflammation ↑SOCS3 ↑ STAT3	↓ Cognitive deficits ↓ Amyloid β peptide	[19]
Anti-allergic	In vitro In vivo	↑NF-κB-miR-183-5p-BTG1 axis	↓ Allergic reactions ↓ Symptoms of atopic dermatitis	[141]
Cardioprotective effect in diabetes-induced ischemic heart disease	In vivo ND-fed and HFD-induced diabetic mice	↓ Ribosomal function ↑ Cardiomyocyte apoptosis	↓ Cardiomyocyte apoptosis ↓ Protective against ischemia/reperfusion injury	[145]
Anti-viral	In vitro In vivo	↓ Viral replication ↓ Lytic gene expression ↓ RNA viruses	↓ Viral load in multiple viruses, including SARS-CoV-2 reduced disease symptoms	[13, 148–152, 154–156]

ER: Endoplasmic Reticulum; HFD: High Fat Diet; ND: Normal Diet; NF-κB: Nuclear Factor Kappa-Light-Chain-Enhancer of Activated B Cells; SOCS3: Suppressor of Cytokine Signaling 3; STAT3: Signal Transducer and Activator of Transcription 3

## Limitations

HHT and its derivatives, such as Omacetaxine Mepesuccinate (OM), hold significant promise for the treatment of various malignancies, but there are several limitations to the current body of research that should be addressed in future studies. Although HHT is generally well-tolerated, the long-term side effects and optimal dosing regimens require further investigation to minimize toxicity and maximize patient quality of life. The pharmacokinetic properties and bioavailability of HHT and its analogs, particularly concerning oral administration, need further refinement to enhance therapeutic outcomes and patient compliance. The patient populations in clinical trials have been heterogeneous, and there is a need for studies in more narrowly defined patient subsets to understand the differential responses to HHT-based therapies and more comprehensive clinical trials are needed to determine the efficacy and safety of HHT in treating non-hematological cancers. HHT has shown potential in non-cancerous conditions such as diabetes, Alzheimer’s disease, and inflammation-related disorders, the research is preliminary, and there is a substantial gap between current knowledge and clinical application. The potential for HHT in combination therapy with other conventional cytotoxic agents has been acknowledged, but optimal combination strategies, sequencing, and maintenance regimens require systematic exploration. Regulatory approval processes for new drugs and their analogs can be lengthy and complex, potentially delaying the availability of improved formulations of HHT and its analogs to patients who could benefit from them.

## Conclusion and future perspectives

HHT has emerged as a promising therapeutic agent in the realm of hematological malignancies, with clinical evidence supporting its efficacy in managing treatment-resistant Chronic Myeloid Leukemia (CML), particularly in cases harboring the challenging BCR–ABL T315I mutation. The possibility of reintroducing tyrosine kinase inhibitors (TKIs) after HHT treatment offers a strategic advantage in clinical scenarios. HHT's remarkable efficacy as a monotherapy or in combination with interferon-alpha (IFN-α) and cytarabine (ara-C) for treating CML in chronic phase (CP) further underscores its therapeutic potential. The impact of HHT, alongside its oral methylated derivative Omacetaxine Mepesuccinate (OM), on leukemia-initiating cells (LICs) has been notable, suggesting that further exploration into combination treatments for newly diagnosed CML patients could be beneficial. For relapsed/refractory acute myeloid leukemia (R/R-AML), high-risk myelodysplastic syndromes (MDS), and MDS/AML, particularly in elderly patients, HHT-based regimens have been well-received, indicating both tolerability and efficacy. However, there is a paucity of clinical data concerning the use of OM in AML and MDS treatment, an area ripe for future research. The exploration of HHT's applications beyond hematological malignancies, particularly its cytotoxic effects against solid tumors such as triple-negative breast cancer, liver, colon, lung, and skin cancers, has opened new avenues for research. The potential for HHT to be integrated into combination, sequential, or maintenance therapy with other anticancer agents is vast, with ongoing research aiming to optimize such regimens. In parallel, HHT's utility is being investigated in other biomedical applications, including antimicrobial, antidiabetic, neuroprotective, and anti-inflammatory treatments. The initial findings are promising, but they necessitate rigorous research to translate these benefits into clinical practice. Moving forward, the development of new HHT analogs with enhanced safety profiles and improved oral bioavailability remains a priority. These endeavors could broaden the spectrum of HHT's clinical applications and offer a more convenient administration route for patients, potentially improving compliance and quality of life. The future of HHT and its derivatives in the treatment of diverse cancers and other diseases looks promising. Continued research is essential to fully harness the therapeutic potentials of HHT, improve patient outcomes, and expand its use in the ever-evolving landscape of medical science.

## Data Availability

Not applicable.

## Abbreviations

AML: Acute myeloid leukemia
Akt: Protein kinase B
allo-HSCT: Allogeneic hematopoietic stem cell transplantation
BAK: Bcl-2 homologous antagonist/killer
Bax: Bcl-2-associated X protein
Bcl-2: B-cell lymphoma 2
BCR–ABL: Breakpoint Cluster Region–Abelson
BP: Blastic phase
Caspases: Cysteine–aspartic proteases
CG: Cytogenetic
CHR: Complete hematologic response
CML: Chronic myeloid leukemia
CP: Chronic phase
CR: Complete remission
Cyt C: Cytochrome c
DLBCL: Diffuse large B-cell lymphoma
DNR: Daunorubicin
EMA: European Medicines Agency
ER: Endoplasmic reticulum
ERK: Extracellular signal-regulated kinase
ERK1/2: Extracellular signal-regulated kinases 1 and 2
FDA: Food and Drug Administration
FLT3: Fms-related tyrosine kinase 3
FLT3–ITD: FMS-like tyrosine kinase 3—internal tandem duplication
HAA: HHT, Aclarubicin, and Cytarabine combination
HFD: High fat diet
Hippo: Signaling pathway
HHT: Homoharringtonine
HSP90: Heat Shock Protein 90
HVA: HHT, Venetoclax, and Azacitidine combination
IFN-α: Interferon alpha
IL-6: Interleukin 6
JAK: Janus kinase
LICs: Leukemia initiating cells
MAPK: Mitogen-activated protein kinase
MCL-1: Myeloid cell leukemia 1
MCyR: Major cytogenetic response
MEK: Mitogen-activated protein kinase kinase
MEK1/2: Mitogen-activated protein kinase kinase 1 and 2
MMP: Mitochondrial membrane potential
Nanog: Stem cell proliferation protein
ND: Normal diet
NF-κB: Nuclear Factor Kappa-Light-Chain-Enhancer of Activated B Cells
NOXA: Phorbol-12-myristate-13-acetate-induced protein 1
OM: Omacetaxine mepesuccinate
OS: Overall survival
PARP: Poly (ADP-ribose) polymerase
Ph +: Philadelphia chromosome positive
PI3K–AKT: Phosphoinositide 3-kinase—protein kinase B
R/R-AML: Refractory/relapsed acute myeloid leukemia
ROS: Reactive oxygen species
SOCS3: Suppressor of cytokine signaling 3
STAT3: Signal transducer and activator of transcription 3
STAT5: Signal transducer and activator of transcription 5
TKI: Tyrosine kinase inhibitor
TNBC: Triple-negative breast cancer
TMEM16A: Transmembrane protein 16A

## References

[1] Swerdlow SH, Campo E, Pileri SA, Harris NL, Stein H, Siebert R, Advani R, Ghielmini M, Salles GA, Zelenetz AD, Jaffe ES (2016). The 2016 revision of the World Health Organization classification of lymphoid neoplasms. Blood.

[2] Keykhaei M, Masinaei M, Mohammadi E, Azadnajafabad S, Rezaei N, Saeedi Moghaddam S, Rezaei N, Nasserinejad M, Abbasi-Kangevari M, Malekpour M-R, Ghamari S-H, Haghshenas R, Koliji K, Kompani F, Farzadfar F (2021). A global, regional, and national survey on burden and Quality of Care Index (QCI) of hematologic malignancies; global burden of disease systematic analysis 1990–2017. Exp Hematol Oncol.

[3] Lin L-T, Hsu W-C, Lin C-C (2014). Antiviral natural products and herbal medicines. J Tradit Complement Med.

[4] Vetrie D, Helgason GV, Copland M (2020). The leukaemia stem cell: similarities, differences and clinical prospects in CML and AML. Nat Rev Cancer.

[5] Hao T, Li-Talley M, Buck A, Chen W (2019). An emerging trend of rapid increase of leukemia but not all cancers in the aging population in the United States. Sci Rep.

[6] Hu Y, Li Q, Hou M, Peng J, Yang X, Xu S (2021). Magnitude and temporal trend of the chronic myeloid leukemia: on the basis of the global burden of disease study 2019. JCO Glob Oncol.

[7] Wu J, Wei B, Shi Y, Lu X, Ding Y, Wang C, Li Y (2019). Homoharringtonine enhances the effect of imatinib on chronic myelogenous leukemia cells by downregulating ZFX. Mol Med Rep.

[8] Stone RM, Donohue KA, Stock W, Hars V, Linker CA, Shea T, DeAngelo DJ, Marcucci G, Bloomfield CD, Larson RA (2009). A phase II study of continuous infusion homoharringtonine and cytarabine in newly diagnosed patients with chronic myeloid leukemia: CALGB study 19804. Cancer Chemother Pharmacol.

[9] Shallis RM, Wang R, Davidoff A., Ma X, & Zeidan AM. Epidemiology of acute myeloid leukemia: Recent progress and enduring challenges. Blood reviews. 2019;36:70–87. 10.1016/j.blre.2019.04.005. 10.1016/j.blre.2019.04.005 31101526

[10] Mi R, Zhao J, Chen L, Wei X, Liu J (2021). Efficacy and safety of homoharringtonine for the treatment of acute myeloid leukemia: a meta-analysis. Clin Lymphoma Myeloma Leuk.

[11] Wang L-b, Wang D-n, Wu L-g, Cao J, Tian J-h, Liu R, Ma R, Yu J-j, Wang J, Huang Q (2021). Homoharringtonine inhibited breast cancer cells growth via miR-18a-3p/AKT/mTOR signaling pathway. Int J Biol Sci.

[12] Quintás-Cardama A, Kantarjian H, Cortes J (2009). Homoharringtonine, omacetaxine mepesuccinate, and chronic myeloid leukemia circa 2009. Cancer.

[13] Dong H-J, Wang Z-H, Meng W, Li C-C, Hu Y-X, Zhou L, Wang X-J (2018). The natural compound homoharringtonine presents broad antiviral activity in vitro and in vivo. Viruses.

[14] Kantarjian HM, O'Brien S, Cortes J (2013). Homoharringtonine/omacetaxine mepesuccinate: the long and winding road to food and drug administration approval. Clin Lymphoma Myeloma Leuk.

[15] Lichota A, Gwozdzinski K (2018). Anticancer activity of natural compounds from plant and marine environment. Int J Mol Sci.

[16] Tang J-f, Li G-l, Zhang T, Du Y-m, Huang S-y, Ran J-h, Li J, Chen D-l (2021). Homoharringtonine inhibits melanoma cells proliferation in vitro and vivo by inducing DNA damage, apoptosis, and G2/M cell cycle arrest. Arch Biochem Biophys.

[17] Guo S, Bai X, Shi S, Deng Y, Kang X, An H (2021). TMEM16A, a homoharringtonine receptor, as a potential endogenic target for lung cancer treatment. Int J Mol Sci.

[18] Plett R, Mellor P, Kendall S, Hammond SA, Boulet A, Plaza K, Vizeacoumar FS, Vizeacoumar FJ, Anderson DH (2022). Homoharringtonine demonstrates a cytotoxic effect against triple-negative breast cancer cell lines and acts synergistically with paclitaxel. Sci Rep.

[19] Jiang X, Wu Q, Zhang C, Wang M (2021). Homoharringtonine inhibits Alzheimer’s disease progression by reducing neuroinflammation via STAT3 signaling in APP/PS1 mice. Neurodegener Dis.

[20] Li X, Wang S, Dai J, Yan L, Zhao S, Wang J, Sun Y (2017). Homoharringtonine prevents surgery-induced epidural fibrosis through endoplasmic reticulum stress signaling pathway. Eur J Pharmacol.

[21] Lü S, Wang J (2014). Homoharringtonine and omacetaxine for myeloid hematological malignancies. J Hematol Oncol.

[22] PubChem. https://pubchem.ncbi.nlm.nih.gov/. Accessed 16 Jan 2023.

[23] WFO. 2023. https://www.worldfloraonline.org/. Accessed 21 Jan 2023.

[24] Hao DC, Xiao PG, Huang B, Ge GB, Yang L (2008). Interspecific relationships and origins of Taxaceae and Cephalotaxaceae revealed by partitioned Bayesian analyses of chloroplast and nuclear DNA sequences. Plant Syst Evol.

[25] Novotny L, Al-Tannak N, Hunakova L (2016). Protein synthesis inhibitors of natural origin for CML therapy: semisynthetic homoharringtonine (Omacetaxine mepesuccinate). Neoplasma.

[26] Vidaković M. Conifers: morphology and variation. Grafičko Zavod Hrvatske. 1991.

[27] Tripp KE (1995). *Cephalotaxus*: the plum yews. Arnoldia.

[28] Silba J (1986) Encyclopaedia coniferae. In: Phytologia memoirs. Vol VIII. Corvallis: Moldenke and Moldenke,

[29] Kantarjian HM, Talpaz M, Santini V, Murgo A, Cheson B, O'Brien SM (2001). Homoharringtonine: history, current research, and future directions. Cancer.

[30] Powell R, Weisleder D, Smith C, Wolff I (1969). Structure of cephalotaxine and related alkaloids. Tetrahedron Lett.

[31] Dai W-J, Dai H-F, Wu J, Liu J, Mei W-L (2009). A new 5-acyl-2-methylpyrrole from the endophytic fungus S20 of *Cephalotaxus hainanensis*. Nat Prod Commun.

[32] Saithong P, Panthavee W, Stonsaovapak S, Li C (2010). Isolation and primary identification of endophytic fungi from *Cephalotaxus mannii* trees. Maejo Int J Sci Technol.

[33] Sakai T, Okumura C, Futamura M, Noda N, Nagae A, Kitamoto C, Kamiya M, Mori Y (2021). Gold (I)-catalyzed cyclization–3-aza-cope–mannich cascade and its application to the synthesis of cephalotaxine. Org Lett.

[34] Lu X, Chen G, Hua H, Dai H, Mei W, Xu Y, Pei Y (2012). Aromatic compounds from endophytic fungus *Colletotrichum* sp. L10 of *Cephalotaxus hainanensis* Li. Fitoterapia.

[35] Xue H, Lu C, Liang L, Shen Y (2012). Secondary metabolites of *Aspergillus* sp. CM9a, an endophytic fungus of *Cephalotaxus mannii*. Rec Nat Prod.

[36] Hu X, Li W, Yuan M, Li C, Liu S, Jiang C, Wu Y, Cai K, Liu Y (2016). Homoharringtonine production by endophytic fungus isolated from *Cephalotaxus hainanensis* Li. World J Microbiol Biotechnol.

[37] Tilaoui M, Mouse HA, Zyad A (2021). Update and new insights on future cancer drug candidates from plant-based alkaloids. Front Pharmacol.

[38] Kim B-S, Kim J-H (2009). Characterization of solvent induced crystalline and amorphous homoharringtonine. Korean J Chem Eng.

[39] Gu X, Chen Y, Lou Y, Zheng J (2019). Separation and characterization of forced degradation products in homoharringtonine injection by UHPLC-Q-TOF-MS. J Pharm Biomed Anal.

[40] Choi YH, Yoo K-P, Kim J (2003). HPLC-electrospray ionization-MS-MS analysis of *Cephalotaxus harringtonia* leaves and enhancement of the extraction efficiency of alkaloids therein by SFE. J Chromatogr Sci.

[41] Nett RS, Guan X, Smith K, Faust AM, Sattely ES, Fischer CR (2018). D2O labeling to measure active biosynthesis of natural products in medicinal plants. AIChE J.

[42] Parry RJ, Chang MN, Schwab JM, Foxman B (1980). Biosynthesis of the *Cephalotaxus* alkaloids: investigations of the early and late stages of cephalotaxine biosynthesis. J Am Chem Soc.

[43] Semmelhack M, Chong B, Jones L (1972). Total synthesis of *Cephalotaxus* alkaloids. J Am Chem Soc.

[44] Weinreb SM, Auerbach J (1975). Total synthesis of the *Cephalotaxus* alkaloids: cephalotaxine, cephalotaxinone, and demethylcephalotaxinone. J Am Chem Soc.

[45] Gouthami P, Chegondi R, Chandrasekhar S (2016). Formal total synthesis of (±)-cephalotaxine and congeners via aryne insertion reaction. Org Lett.

[46] Ju X, Beaudry CM (2019). Total synthesis of (−)-cephalotaxine and (−)-homoharringtonine via furan oxidation-transannular mannich cyclization. Angew Chem.

[47] Dang F-F, Wang C-C, Han F, Zhang Z-W (2021). Synthesis of the ester side chains of homoharringtonine and harringtonine using lactones as building blocks. Synth Commun.

[48] Hiranuma S, Hudlicky T (1982). Synthesis of homoharringtonine and its derivative by partial esterification of cephalotaxine. Tetrahedron Lett.

[49] Esmieu WR, Worden SM, Catterick D, Wilson C, Hayes CJ (2008). A formal synthesis of (-)-cephalotaxine. Org Lett.

[50] Jeon H, Chung Y, Kim S (2019). Proline ester enolate claisen rearrangement and formal total synthesis of (−)-cephalotaxine. J Org Chem.

[51] Yang C, Zhang H, Chen M, Wang S, Qian R, Zhang L, Huang X, Wang J, Liu Z, Qin W (2022). A survey of optimal strategy for signature-based drug repositioning and an application to liver cancer. Elife.

[52] Levy V, Zohar S, Bardin C, Vekhoff A, Chaoui D, Rio B, Legrand O, Sentenac S, Rousselot P, Raffoux E (2006). A phase I dose-finding and pharmacokinetic study of subcutaneous semisynthetic homoharringtonine (ssHHT) in patients with advanced acute myeloid leukaemia. Br J Cancer.

[53] Khazir J, Mir BA, Pilcher L, Riley DL (2014). Role of plants in anticancer drug discovery. Phytochem Lett.

[54] Darwiche N, El-Banna S, Gali-Muhtasib H (2007). Cell cycle modulatory and apoptotic effects of plant-derived anticancer drugs in clinical use or development. Expert Opin Drug Discov.

[55] Wetzler M, Segal D (2011). Omacetaxine as an anticancer therapeutic: what is old is new again. Curr Pharm Des.

[56] Zhang T, Shen S, Zhu Z, Lu S, Yin X, Zheng J, Jin J (2016). Homoharringtonine binds to and increases myosin-9 in myeloid leukaemia. Br J Pharmacol.

[57] Muralidharan A, Scott JJX, Joseph LD, Jeyabalan S (2021). Effect of homoharringtonine as a combined regimen for acute myeloid leukemia. J Pharmacol Pharmacother.

[58] Zhang Y, Li N, Chang Z, Wang H, Pei H, Zhang D, Zhang Q, Huang J, Guo Y, Zhao Y (2022). The metabolic signature of AML cells treated with homoharringtonine. Front Oncol.

[59] DiNardo CD, Wei AH (2020). How I treat acute myeloid leukemia in the era of new drugs. Blood.

[60] Reikvam H, Hatfield KJ, Kittang AO, Hovland R, Bruserud Ø (2011). Acute myeloid leukemia with the t (8; 21) translocation: clinical consequences and biological implications. J Biomed Biotechnol.

[61] Zhang W, Lu Y, Zhen T, Chen X, Zhang M, Liu P, Weng X, Chen B, Wang Y (2019). Homoharringtonine synergy with oridonin in treatment of t (8; 21) acute myeloid leukemia. Front Med.

[62] Lai C, Doucette K, Norsworthy K (2019). Recent drug approvals for acute myeloid leukemia. J Hematol Oncol.

[63] Watanabe D, Nogami A, Okada K, Akiyama H, Umezawa Y, Miura O (2019). FLT3-ITD activates RSK1 to enhance proliferation and survival of AML cells by activating mTORC1 and eIF4B cooperatively with PIM or PI3K and by inhibiting bad and BIM. Cancers.

[64] Ofran Y, Tallman MS, Rowe JM (2016). How I treat acute myeloid leukemia presenting with preexisting comorbidities. Blood J Am Soc Hematol.

[65] Kantarjian H, Kadia T, DiNardo C, Daver N, Borthakur G, Jabbour E, Garcia-Manero G, Konopleva M, Ravandi F (2021). Acute myeloid leukemia: current progress and future directions. Blood Cancer J.

[66] Bohlander SK (2020). A new kid on the block for acute myeloid leukemia treatment? Homoharringtonine interferes with key pathways in acute myeloid leukemia cells. Haematologica.

[67] Zhou J-Y, Chen D-L, Shen Z-S, Koeffler HP (1990). Effect of homoharringtonine on proliferation and differentiation of human leukemic cells in vitro. Can Res.

[68] He J, Li L, Zhu J, Zhao Y, Wu W, Zheng Y, Zheng G, Zheng W, Zhu X, Huang H (2013). Homoharringtonine in combination with cytarabine and etoposide for induction therapy in patients with de novo acute myelogenous leukemia. Blood.

[69] Tang R, Faussat A-M, Majdak P, Marzac C, Dubrulle S, Marjanovic Z, Legrand O, Marie J-P (2006). Semisynthetic homoharringtonine induces apoptosis via inhibition of protein synthesis and triggers rapid myeloid cell leukemia-1 down-regulation in myeloid leukemia cells. Mol Cancer Ther.

[70] Yuan F, Li D, Li G, Cheng C, Wei X (2022). Synergistic efficacy of homoharringtonine and venetoclax on acute myeloid leukemia cells and the underlying mechanisms. Ann Transl Med.

[71] Wang L, You L-S, Ni W-M, Ma Q-L, Tong Y, Mao L-P, Qian J-J, Jin J (2013). β-Catenin and AKT are promising targets for combination therapy in acute myeloid leukemia. Leuk Res.

[72] Cao J, Feng H, Ding NN, Qy Wu, Chen C, Niu MS, Chen W, Qiu TT, Zhu HH, Xu KL (2016). Homoharringtonine combined with aclarubicin and cytarabine synergistically induces apoptosis in t (8; 21) leukemia cells and triggers caspase-3-mediated cleavage of the AML1-ETO oncoprotein. Cancer Med.

[73] Zhang J, Geng H, Liu L, Zhang H (2019). Synergistic cytotoxicity of homoharringtonine and etoposide in acute myeloid leukemia cells involves disrupted antioxidant defense. Cancer Manag Res.

[74] Wu Z, Zhuang H, Yu Q, Zhang X, Jiang X, Lu X, Xu Y, Yang L, Wu B, Ma A (2019). Homoharringtonine combined with the heat shock protein 90 inhibitor IPI504 in the treatment of FLT3-ITD acute myeloid leukemia. Transl Oncol.

[75] Li X, Yin X, Wang H, Huang J, Yu M, Ma Z, Li C, Zhou Y, Yan X, Huang S (2017). The combination effect of homoharringtonine and ibrutinib on FLT3-ITD mutant acute myeloid leukemia. Oncotarget.

[76] Wang F, Huang J, Guo T, Zheng Y, Zhang L, Zhang D, Wang F, Naren D, Cui Y, Liu X (2021). Homoharringtonine synergizes with quizartinib in FLT3-ITD acute myeloid leukemia by targeting FLT3-AKT-c-Myc pathway. Biochem Pharmacol.

[77] Shi Y, Ye J, Yang Y, Zhao Y, Shen H, Ye X, Xie W (2021). The basic research of the combinatorial therapy of ABT-199 and homoharringtonine on acute myeloid leukemia. Front Oncol.

[78] Klanova M, Andera L, Brazina J, Svadlenka J, Benesova S, Soukup J, Prukova D, Vejmelkova D, Jaksa R, Helman K (2016). Targeting of BCL2 family proteins with ABT-199 and homoharringtonine reveals BCL2-and MCL1-dependent subgroups of diffuse large B-cell lymphoma targeting of BCL2 proteins in diffuse large B-cell lymphoma. Clin Cancer Res.

[79] Yu G, Xu N, Huang F, Fan Z, Liu H, Shi P, Zhou H, Wang Z, Zhang Y, Liu Q (2020). Combination of homoharringtonine with venetoclax and azacitidine excerts better treatment response in relapsed/refractory acute myeloid leukemia. Blood.

[80] Yu W, Mao L, Qian J, Qian W, Meng H, Mai W, Tong H, Tong Y, Jin J (2013). Homoharringtonine in combination with cytarabine and aclarubicin in the treatment of refractory/relapsed acute myeloid leukemia: a single-center experience. Ann Hematol.

[81] Jin J, Wang J-X, Chen F-F, Wu D-P, Hu J, Zhou J-F, Hu J-D, Wang J-M, Li J-Y, Huang X-J (2013). Homoharringtonine-based induction regimens for patients with de-novo acute myeloid leukaemia: a multicentre, open-label, randomised, controlled phase 3 trial. Lancet Oncol.

[82] Zhu H-H, Jiang H, Jiang Q, Jia J-S, Qin Y-Z, Huang X-J (2016). Homoharringtonine, aclarubicin and cytarabine (HAA) regimen as the first course of induction therapy is highly effective for acute myeloid leukemia with t (8; 21). Leuk Res.

[83] Li Y, Zhang B, Liu M, Zhang X, Shi D, Guo L, Duan J, Zhou X, Zhu H, Zhang Q (2018). Further study of influence of *Panax notoginseng* on intestinal absorption characteristics of triptolide and tripterine in rats with *Tripterygium wilfordii*. Pharmacogn Mag.

[84] Kabat GC, Wu JW, Moore SC, Morton LM, Park Y, Hollenbeck AR, Rohan TE (2013). Lifestyle and dietary factors in relation to risk of chronic myeloid leukemia in the NIH-AARP diet and health study risk factors for chronic myeloid leukemia. Cancer Epidemiol Biomark Prev.

[85] Strom SS, Yamamura Y, Kantarijian HM, Cortes-Franco JE (2009). Obesity, weight gain, and risk of chronic myeloid leukemia. Cancer Epidemiol Biomark Prev.

[86] Thompson PA, Kantarjian HM (2015). Cortes JE Diagnosis and treatment of chronic myeloid leukemia in 2015. Mayo Clin Proc.

[87] Kujawski LA, Talpaz M (2007). The role of interferon-alpha in the treatment of chronic myeloid leukemia. Cytokine Growth Factor Rev.

[88] Rosshandler Y, Shen AQ, Cortes J, Khoury HJ (2016). Omacetaxine mepesuccinate for chronic myeloid leukemia. Expert Rev Hematol.

[89] Cortes J, Lipton JH, Rea D, Digumarti R, Chuah C, Nanda N, Benichou A-C, Craig AR, Michallet M, Nicolini FE (2012). Phase 2 study of subcutaneous omacetaxine mepesuccinate after TKI failure in patients with chronic-phase CML with T315I mutation. Blood J Am Soc Hematol.

[90] Rossari F, Minutolo F, Orciuolo E (2018). Past, present, and future of Bcr-Abl inhibitors: from chemical development to clinical efficacy. J Hematol Oncol.

[91] Cortes J, Digumarti R, Parikh P, Wetzler M, Lipton J, Hochhaus A, Craig A, Benichou AC, Nicolini F, Kantarjian H (2013). Phase 2 study of subcutaneous omacetaxine mepesuccinate for chronic-phase chronic myeloid leukemia patients resistant to or intolerant of tyrosine kinase inhibitors. Am J Hematol.

[92] Granatowicz A, Piatek CI, Moschiano E, El-Hemaidi I, Armitage JD, Akhtari M (2015). An overview and update of chronic myeloid leukemia for primary care physicians. Korean J Fam Med.

[93] Maiti A, Cortes J, Ferrajoli A, Estrov Z, Borthakur G, Garcia-Manero G, Jabbour E, Ravandi F, O’Brien S, Kantarjian H (2017). Phase II trial of homoharringtonine with imatinib in chronic, accelerated, and blast phase chronic myeloid leukemia. Leuk Lymphoma.

[94] Winer ES, DeAngelo DJ (2018). A review of omacetaxine: a chronic myeloid leukemia treatment resurrected. Oncol Ther.

[95] Visani G, Russo D, Ottaviani E, Tosi P, Damiani D, Michelutti A, Manfroi S, Baccarani M, Tura S (1997). Effects of homoharringtonine alone and in combination with alpha interferon and cytosine arabinoside on ‘in vitro’growth and induction of apoptosis in chronic myeloid leukemia and normal hematopoietic progenitors. Leukemia.

[96] Okabe S, Tauchi T, Tanaka Y, Katagiri S, Kitahara T, Ohyashiki K (2013). Activity of omacetaxine mepesuccinate against ponatinib-resistant BCR-ABL-positive cells. Blood.

[97] Chen Y, Hu Y, Michaels S, Segal D, Brown D, Li S (2009). Inhibitory effects of omacetaxine on leukemic stem cells and BCR-ABL-induced chronic myeloid leukemia and acute lymphoblastic leukemia in mice. Leukemia.

[98] Li Y-F, Liu X, Liu D-S, Din B-H, Zhu J-B (2009). The effect of homoharringtonine in patients with chronic myeloid leukemia who have failed or responded suboptimally to imatinib therapy. Leuk Lymphoma.

[99] Chen R, Gandhi V, Plunkett W (2006). A sequential blockade strategy for the design of combination therapies to overcome oncogene addiction in chronic myelogenous leukemia. Can Res.

[100] Nguyen T, Parker R, Zhang Y, Hawkins E, Kmieciak M, Craun W, Grant S (2018). Homoharringtonine interacts synergistically with bortezomib in NHL cells through MCL-1 and NOXA-dependent mechanisms. BMC Cancer.

[101] Xie C, Tang A-P (2018). Combined effect of Bortezomib and Homoharringtonine on K562 cells and their mechanisms. Zhongguo shi yan xue ye xue za zhi.

[102] O'Brien S, Kantarjian H, Keating M, Beran M, Koller C, Robertson L, Hester J, Rios M, Andreeff M, Talpaz M (1995). Homoharringtonine therapy induces responses in patients with chronic myelogenous leukemia in late chronic phase. Blood.

[103] O’Brien S, Kantarjian H, Koller C, Feldman E, Beran M, Andreeff M, Giralt S, Cheson B, Keating M, Freireich E (1999). Sequential homoharringtonine and interferon-α in the treatment of early chronic phase chronic myelogenous leukemia. Blood J Am Soc Hematol.

[104] O'Brien S, Talpaz M, Cortes J, Shan J, Giles FJ, Faderl S, Thomas D, Garcia-Manero G, Mallard S, Beth Rios M (2002). Simultaneous homoharringtonine and interferon-α in the treatment of patients with chronic-phase chronic myelogenous leukemia. Cancer.

[105] Kantarjian HM, Talpaz M, Smith TL, Cortes J, Giles FJ, Rios MB, Mallard S, Gajewski J, Murgo A, Cheson B (2000). Homoharringtonine and low-dose cytarabine in the management of late chronic-phase chronic myelogenous leukemia. J Clin Oncol.

[106] Marin D, Kaeda JS, Andreasson C, Saunders SM, Bua M, Olavarria E, Goldman JM, Apperley JF (2005). Phase I/II trial of adding semisynthetic homoharringtonine in chronic myeloid leukemia patients who have achieved partial or complete cytogenetic response on imatinib. Cancer.

[107] Quintás-Cardama A, Kantarjian H, Garcia-Manero G, O'Brien S, Faderl S, Estrov Z, Giles F, Murgo A, Ladie N, Verstovsek S (2007). Phase I/II study of subcutaneous homoharringtonine in patients with chronic myeloid leukemia who have failed prior therapy. Cancer.

[108] O'Brien S, Giles F, Talpaz M, Cortes J, Rios MB, Shan J, Thomas D, Andreeff M, Kornblau S, Faderl S (2003). Results of triple therapy with interferon-alpha, cytarabine, and homoharringtonine, and the impact of adding imatinib to the treatment sequence in patients with Philadelphia chromosome–positive chronic myelogenous leukemia in early chronic phase. Cancer Interdiscip Int J Am Cancer Soc.

[109] Chen R, Guo L, Chen Y, Jiang Y, Wierda WG, Plunkett W (2011). Homoharringtonine reduced Mcl-1 expression and induced apoptosis in chronic lymphocytic leukemia. Blood J Am Soc Hematol.

[110] Jin Y, Lu Z, Cao K, Zhu Y, Chen Q, Zhu F, Qian C, Pan J (2010). The antitumor activity of homoharringtonine against human mast cells harboring the KIT D816V mutation. Mol Cancer Ther.

[111] Sah SK, Shah S, Tiwari SB, Poudel BS, Singh B, Sharma P, Acharya SS, Murarka H, Thapaliya S, Shrestha A (2022). Essential thrombocythemia among patients with myeloproliferative neoplasms in haematology unit of a Tertiary Care Centre: a descriptive cross-sectional study. JNMA J Nepal Med Assoc.

[112] Li Y, Zhu J, Ding B (2013). Homoharringtonine is an effective therapy for patients with polycythemia vera or essential thrombocythemia who have failed or were intolerant to hydroxycarbamide or interferon-α therapy. Int J Clin Oncol.

[113] Feng Y, Spezia M, Huang S, Yuan C, Zeng Z, Zhang L, Ji X, Liu W, Huang B, Luo W, Liu B, Lei Y, Du S, Vuppalapati A, Luu HH, Haydon RC, He TC, Ren G (2018). Breast cancer development and progression: risk factors, cancer stem cells, signaling pathways, genomics, and molecular pathogenesis. Genes Dis.

[114] Urzì AG, Tropea E, Gattuso G, Spoto G, Marsala G, Calina D, Libra M, Falzone L (2023). Ketogenic diet and breast cancer: recent findings and therapeutic approaches. Nutrients.

[115] Yakhni M, Briat A, El Guerrab A, Furtado L, Kwiatkowski F, Miot-Noirault E, Cachin F, Penault-Llorca F, Radosevic-Robin N (2019). Homoharringtonine, an approved anti-leukemia drug, suppresses triple negative breast cancer growth through a rapid reduction of anti-apoptotic protein abundance. Am J Cancer Res.

[116] Chen P, Wen X, Wang B, Hou D, Zou H, Yuan Q, Yang H, Xie J, Huang H (2018). PI3K/Akt inhibitor LY294002 potentiates homoharringtonine antimyeloma activity in myeloma cells adhered to stromal cells and in SCID mouse xenograft. Ann Hematol.

[117] Cao W, Liu Y, Zhang R, Zhang B, Wang T, Zhu X, Mei L, Chen H, Zhang H, Ming P (2015). Homoharringtonine induces apoptosis and inhibits STAT3 via IL-6/JAK1/STAT3 signal pathway in Gefitinib-resistant lung cancer cells. Sci Rep.

[118] Weng T-Y, Wu HF, Li C-Y, Hung Y-H, Chang Y-W, Chen Y-L, Hsu H-P, Chen Y-H, Wang C-Y, Chang J-Y (2018). Homoharringtonine induced immune alteration for an efficient anti-tumor response in mouse models of non-small cell lung adenocarcinoma expressing Kras mutation. Sci Rep.

[119] Wilkoff LJ, Dulmadge EA, Vasanthakumar G, Donahue JP (1993). Etoposide-resistant human colon and lung adenocarcinoma cell lines exhibit sensitivity to homoharringtonine. Cancer Chemother Pharmacol.

[120] Wang H, Wang R, Huang D, Li S, Gao B, Kang Z, Tang B, Xie J, Yan F, Liang R (2021). Homoharringtonine exerts anti-tumor effects in hepatocellular carcinoma through activation of the hippo pathway. Front Pharmacol.

[121] Zhu M, Gong Z, Wu Q, Su Q, Yang T, Yu R, Xu R, Zhang Y (2020). Homoharringtonine suppresses tumor proliferation and migration by regulating EphB4-mediated β-catenin loss in hepatocellular carcinoma. Cell Death Dis.

[122] Yang Y, Yu Q, Hu L, Dai B, Qi R, Chang Y, Zhang Q, Zhang Z, Li Y, Zhang X (2022). Enantioselective semisynthesis of novel cephalotaxine esters with potent antineoplastic activities against leukemia. Eur J Med Chem.

[123] Shi X, Zhu M, Gong Z, Yang T, Yu R, Wang J, Zhang Y (2020). Homoharringtonine suppresses LoVo cell growth by inhibiting EphB4 and the PI3K/AKT and MAPK/EKR1/2 signaling pathways. Food Chem Toxicol.

[124] Qu M, Li J, Yuan L (2021). Uncovering the action mechanism of homoharringtonine against colorectal cancer by using network pharmacology and experimental evaluation. Bioengineered.

[125] Park M, Kwon HJ, Kim SH (2019). Homoharringtonine induces apoptosis in human colorectal carcinoma HCT116 cells via downregulation of Wnt/β-catenin signaling cascade. Bull Korean Chem Soc.

[126] Beranova L, Pombinho AR, Spegarova J, Koc M, Klanova M, Molinsky J, Klener P, Bartunek P, Andera L (2013). The plant alkaloid and anti-leukemia drug homoharringtonine sensitizes resistant human colorectal carcinoma cells to TRAIL-induced apoptosis via multiple mechanisms. Apoptosis.

[127] Wu D, Jin Y, Xing Y, Abate MD, Abbasian M, Abbasi-Kangevari M, Abbasi-Kangevari Z, Abd-Allah F, Abdelmasseh M, Abdollahifar MA, Abdulah DM (2023). Global, regional, and national incidence of six major immune-mediated inflammatory diseases: findings from the global burden of disease study 2019. EClinicalMedicine.

[128] Kumari R, Negi M, Thakur P, Mahajan H, Raina K, Sharma R, Singh R, Anand V, Ming LC, Goh KW, Calina D, Sharifi-Rad J, Chaudhary A (2023). *Saussurea costus* (Falc.) Lipsch.: a comprehensive review of its pharmacology, phytochemicals, ethnobotanical uses, and therapeutic potential. Naunyn Schmiedebergs Arch Pharmacol.

[129] Yuan G, Wahlqvist ML, He G, Yang M, Li D (2006). Natural products and anti-inflammatory activity. Asia Pac J Clin Nutr.

[130] Tagboto S, Townson S (2001). Antiparasitic properties of medicinal plants and other naturally occurring products.

[131] Krstin S, Mohamed T, Wang X, Wink M (2016). How do the alkaloids emetine and homoharringtonine kill trypanosomes? An insight into their molecular modes of action. Phytomedicine.

[132] Anand R, Gill K, Mahdi A (2014). Therapeutics of Alzheimer’s disease: past, present and future. Neuropharmacology.

[133] Essa MM, Vijayan RK, Castellano-Gonzalez G, Memon MA, Braidy N, Guillemin GJ (2012). Neuroprotective effect of natural products against Alzheimer’s disease. Neurochem Res.

[134] Shal B, Ding W, Ali H, Kim YS, Khan S (2018). Anti-neuroinflammatory potential of natural products in attenuation of Alzheimer’s disease. Front Pharmacol.

[135] Korolev IO (2014). Alzheimer’s disease: a clinical and basic science review. Med Stud Res J.

[136] Nettis E, Colanardi M, Ferrannini A, Tursi A (2005). Antihistamines as important tools for regulating inflammation. Curr Med Chem Anti-Inflamm Anti-Allergy Agents.

[137] Brito F, Lima L, Ramos M, Nakamura M, Cavalher-Machado S, Siani A, Henriques M, Sampaio A (2007). Pharmacological study of anti-allergic activity of *Syzygium cumini* (L.) Skeels. Braz J Med Biol Res.

[138] Bakhotmah BA, Alzahrani HA (2010). Self-reported use of complementary and alternative medicine (CAM) products in topical treatment of diabetic foot disorders by diabetic patients in Jeddah, Western Saudi Arabia. BMC Res Notes.

[139] Harvey A (2000). Strategies for discovering drugs from previously unexplored natural products. Drug Discov Today.

[140] Bellik Y, Hammoudi S, Abdellah F, Iguer-Ouada M, Boukraa L (2012). Phytochemicals to prevent inflammation and allergy. Recent Pat Inflamm Allergy Drug Discov.

[141] Kim M, Jo H, Kwon Y, Kim Y, Jung HS, Jeoung D (2020). Homoharringtonine inhibits allergic inflammations by regulating NF-κB-miR-183-5p-BTG1 axis. Front Pharmacol.

[142] Ong KL, Stafford LK, McLaughlin SA, Boyko EJ, Vollset SE, Smith AE, Dalton BE, Duprey J, Cruz JA, Hagins H, Lindstedt PA (2023). Global, regional, and national burden of diabetes from 1990 to 2021, with projections of prevalence to 2050: a systematic analysis for the Global Burden of Disease Study 2021. Lancet.

[143] Ye Y, Perez-Polo JR, Aguilar D, Birnbaum Y (2011). The potential effects of anti-diabetic medications on myocardial ischemia–reperfusion injury. Basic Res Cardiol.

[144] Severino P, D'Amato A, Netti L, et al. Myocardial Ischemia and Diabetes Mellitus: Role of Oxidative Stress in the Connection between Cardiac Metabolism and Coronary Blood Flow. J Diabetes Res. 2019:9489826.10.1155/2019/9489826. Accessed 4 Apr 2019. 10.1155/2019/9489826 PMC6476021 31089475

[145] Zhao S, Xia Y, Zhang F, Xiong Z, Li Y, Yan W, Chen X, Wang W, Wang H, Gao E (2017). Nucleostemin dysregulation contributes to ischemic vulnerability of diabetic hearts: role of ribosomal biogenesis. J Mol Cell Cardiol.

[146] Locarnini SA, Yuen L (2010). Molecular genesis of drug-resistant and vaccine-escape HBV mutants. Antiviral Ther.

[147] Christou L (2011). The global burden of bacterial and viral zoonotic infections. Clin Microbiol Infect.

[148] Kim J-E, Song Y-J (2019). Anti-varicella-zoster virus activity of cephalotaxine esters in vitro. J Microbiol.

[149] Romero MR, Serrano MA, Efferth T, Alvarez M, Marin JJ (2007). Effect of cantharidin, cephalotaxine and homoharringtonine on” in vitro” models of Hepatitis B Virus (HBV) and Bovine Viral Diarrhoea Virus (BVDV) replication. Planta Med.

[150] Andersen PI, Krpina K, Ianevski A, Shtaida N, Jo E, Yang J, Koit S, Tenson T, Hukkanen V, Anthonsen MW (2019). Novel antiviral activities of obatoclax, emetine, niclosamide, brequinar, and homoharringtonine. Viruses.

[151] Li C-C, Wang X-J (2020). Three kinds of treatment with Homoharringtonine, Hydroxychloroquine or shRNA and their combination against coronavirus PEDV in vitro. Virol J.

[152] Zhang J-W, Wang H, Liu J, Ma L, Hua R-H, Bu Z-G (2021). Generation of a stable GFP-reporter Zika virus system for high-throughput screening of Zika virus inhibitors. Virol Sin.

[153] Alvandi F, Kwitkowski VE, Ko C-W, Rothmann MD, Ricci S, Saber H, Ghosh D, Brown J, Pfeiler E, Chikhale E (2014). US Food and Drug Administration approval summary: omacetaxine mepesuccinate as treatment for chronic myeloid leukemia. Oncologist.

[154] Ma H, Wen H, Qin Y, Wu S, Zhang G, Wu C-I, Cai Q (2022). Homo-harringtonine, highly effective against coronaviruses, is safe in treating COVID-19 by nebulization. Sci China Life Sci.

[155] Choy K, Wong AY-L, Kaewpreedee P, Sia SF, Chen D, Hui KPY, Chu DKW, Chan MCW, Cheung PP-H, Huang X, Peiris M, Yen HL (2020). Remdesivir, lopinavir, emetine, and homoharringtonine inhibit SARS-CoV-2 replication in vitro. Antiviral Res.

[156] Boozari M, Hosseinzadeh H (2021). Natural products for COVID-19 prevention and treatment regarding to previous coronavirus infections and novel studies. Phytother Res.

